# Genotypic and phenotypic characteristics of CXCR4-using transmitted/founder HIV-1 envelope glycoproteins

**DOI:** 10.1128/jvi.00067-26

**Published:** 2026-05-14

**Authors:** Dorine Martres, Théo Montagné, Sandra Pani, Nicolas Jeanne, Marie Armani-Tourret, Mary Requena, Camille Vellas, Stéphanie Raymond, Valérie Lorin, Yohan Gallois, Bénédicte Puissant-Lubrano, Hugo Mouquet, Pierre Delobel, Jacques Izopet, Bernard Lagane

**Affiliations:** 1 Infinity – Toulouse Institute for Infectious and Inflammatory Diseases, INSERM UMR 1291, CNRS UMR 5051, University of Toulouse, Toulouse, France; 2 Laboratoire de virologie, CHU de Toulouse 36760 , Toulouse, France; 3 Institut Pasteur, Université Paris Cité, Humoral Immunology Unit 555089, Paris, France; 4 Service d’ORL, Otoneurologie et ORL Pédiatrique, CHU de Toulouse 36760 , Toulouse, France; 5 Service des Maladies Infectieuses et Tropicales, CHU de Toulouse 36760 , Toulouse, France; Icahn School of Medicine at Mount Sinai, New York, New York, USA

**Keywords:** CXCR4, broadly neutralizing antibodies, primary HIV infection, envelope glycoprotein, viral tropism, HIV transmission

## Abstract

The characteristics of HIV-1 envelope glycoproteins (Env) that initiate infection remain incompletely understood. In the primary HIV infection (PHI) stage, Envs using CCR5 as entry coreceptor (R5-tropic Envs) are much more frequent than those using CXCR4, alone or in addition to CCR5 (X4 or R5X4 Envs). However, a comprehensive analysis of the genetic identity and functional properties of CXCR4-using Envs during PHI is lacking to attest whether they are less transmissible than R5 Envs. Here, Env populations in the plasma of 22 recently infected individuals with R5 or CXCR4-using viruses were characterized by single-genome amplification. Mathematical modeling of HIV evolution, phylogenetic analyses, and viral quasi-species complexity indices showed that infection with CXCR4-using viruses is most often initiated by one, occasionally two variants, similar to R5 viruses. After transmission, the diversity and complexity of CXCR4-using and R5 Env populations increase similarly during PHI, with mutations accumulating in preferential regions of the gp120 portion of Env, but not in gp41. Both Env types also shared similar traits influencing viral transmission (variable loop length, N-glycosylations) and T-cell– but non-macrophage–tropism. However, CXCR4-using Envs exhibited specific sensitivity profiles to neutralization by soluble CD4 and broadly neutralizing antibodies (bNAbs), suggesting differences in epitope exposure and conformational dynamics compared to R5 Envs. These results, therefore, indicate that CXCR4-using viruses are equipped to initiate infection and, like R5 viruses, undergo a genetic bottleneck during transmission. The specific antigenicity of transmitted CXCR4-using Envs could help improve protocols using bNAbs for passive immunization and HIV vaccine development.

**IMPORTANCE:**

Transmitted/founder (T/F) viruses are predominantly R5-tropic, and only on rare occasions have T/F viruses that use CXCR4 been reported in the literature. Consistently, R5 viruses account for over 90% of the viruses present during primary HIV infection (PHI). However, it remains unclear whether CXCR4-using Envs, unlike R5 Envs, lack key properties for virus transmission and/or persistence in the new host. Here, we found that transmitted CXCR4-using Envs shared with R5 Envs common genetic and functional determinants, demonstrating their potential to transmit infection. So, the low prevalence of CXCR4-using viruses among donors probably explains why they are infrequently transmitted. As with R5 viruses, a genetic bottleneck occurs during transmission of CXCR4-using viruses, suggesting that the barriers determining this bottleneck are independent of viral tropism. Remarkably, transmitted CXCR4-using Envs exhibit distinct antigenic properties compared to R5 Envs, which could be leveraged to improve broadly neutralizing antibody-based preventive and therapeutic vaccine strategies.

## INTRODUCTION

A better understanding of the determinants of HIV-1 for its transmission and the establishment of a new infection can help the development of preventive and therapeutic vaccines. An ongoing question is whether particular properties of the viral envelope glycoprotein (Env) are required for the transmission of infection. Env allows HIV-1 entry into host immune cells such as CD4+ T lymphocytes (CD4TL) and macrophages (1). It consists of two non-covalently linked subunits, gp120 and gp41, which assemble into a trimer on the virus surface. Gp120, the Env surface subunit consisting of five variable loops (V1–5) separated by five conserved regions (C1–5), initiates viral entry by interacting with the CD4 cell receptor. This interaction induces conformational changes in gp120 that expose binding sites to chemokine coreceptors, CCR5 or CXCR4 (2). Interaction with CD4 leads the Env trimer to change from a closed to an open conformation, from which the three Env membrane subunits, gp41, are released to trigger viral and cell membrane fusion (3, 4). Env is the prime target of antibody-mediated immunity, which develops within a few weeks after transmission (5, 6). Host antibodies exert a selective pressure on Env and drive its diversification in viral quasi-species throughout the infection. In some individuals, broadly neutralizing antibodies (bNAbs), active against a broad range of HIV-1 variants, can emerge after years of infection (7). bNAbs hold great promise for HIV-1 prevention and therapy and are currently in clinical trials (8, 9).

In the primary infection stage (PHI), viruses using CCR5 (R5-tropic viruses) are more frequent than viruses using CXCR4 (10, 11). Phenotypic tropism assays indicated that the proportion of individuals with CXCR4-using viruses during PHI ranges from 1% to 8%, depending on the cohort, viral subtype, and possibly the mode of transmission (12–17). In most cases, these viruses were R5X4/dual-tropic viruses (i.e., they can use both CXCR4 and CCR5), while pure X4-tropic viruses (i.e., using CXCR4 exclusively) were much rarer. Additionally, homozygous individuals for the CCR5Δ32 mutation are highly protected against HIV-1 infection (18, 19), illustrating that initiation of infection via CXCR4 only is infrequent. Consistently, the majority of transmitted/founder (T/F) Envs that have been inferred from phylogenetic analysis of single Env amplicons from early PHI are also R5-tropic (20, 21). However, the reasons why CXCR4-using viruses are infrequently transmitted remain unclear. An obvious hypothesis would be that R5 Envs but not CXCR4-using (X4/R5X4) Envs have characteristics that favor transmission. Actually, transmission of HIV-1 is characterized by a genetic bottleneck such that only one or a few variants of the donor initiate infection (20–24). Analysis of Envs in transmission pairs indicated that T/F viruses were minority variants in the blood or the genital tract of the donor (22, 25), suggesting that T/F Envs harbor characteristics that increase the probability of transmission. Derdeyn et al. showed that Envs in recently sexually infected individuals have shorter V1/V2 loops and are less N-glycosylated, compared with Envs in the transmitting partners, and were more sensitive to neutralization by antibodies from the donor (26). These observations were later reported for some HIV-1 subtypes but not for others (27–32). Others have proposed that transmission does not necessarily select for Envs with particular V1/V2 length and N-glycosylation number, but both characteristics, regardless of viral subtype, increase over time in infection in response to the selective pressure of host humoral immunity (33). Data have since confirmed that the V2 loop is a mutation hotspot in T/F Envs to escape the first neutralizing antibodies (NAbs) that appear in the host (34).

It has also been proposed that T/F Envs share specific receptor-binding properties that increase viral fitness and hence transmission of infection. Specific sequence motifs near the CCR5-binding site have been described in Envs from early infection (35). T/F and chronic Envs have also been shown to differentially interact with distinct CCR5 conformations (31, 36–38), although they were similarly dependent on CCR5 levels (39, 40). T/F and chronic Envs also seem to utilize CD4 with the same efficiency and are equally sensitive to soluble CD4 (sCD4) inhibition (20, 31, 41). Actually, T/F viruses share with the majority of chronic-phase viruses the property of having a weak ability to infect cells that express low levels of CD4 (31, 39, 41), including macrophages (M) (21, 36, 42), identifying them as T-/non-M-tropic viruses. This is reflected in increased resistance of viruses to neutralization by sCD4, compared to M-tropic viruses (31, 42), which has been explained by the fact that non-M-tropic viruses adopt a more closed conformation where the CD4-binding site is less exposed (43). Recent structural data have shown, however, that things are more complex with T/F Envs, which, depending on the viral strain, can adopt a variety of conformations, some of which are incompletely closed (44). Interestingly, the authors also showed that Env conformation tended to be more closed as infection progresses post-transmission and was intimately linked to neutralization sensitivity, in particular to sCD4 and bNAbs (44). Other characteristics of T/F Envs have been proposed to favor transmission; for example, enhanced Env expression or virus infectivity and replication, but with contradictory results (28, 30, 40, 45, 46). Increased resistance of T/F viruses to type-I interferons has also been shown in some articles, but not in others (28, 30, 45, 47, 48).

To date, however, it is unknown to what extent these characteristics, thought to influence transmission of R5 Envs, are shared by X4/R5X4 Envs. Actually, studies have suggested mechanisms that specifically inhibit transmission of CXCR4-using Envs (49, 50). Molecules that bind CXCR4, including the receptor’s natural chemokine SDF-1/CXCL12, and thereby inhibit viral entry, have been identified at sites of virus transmission or in transmission fluids (51–54). It has also been shown that some target cells present at transmission sites are less permissive to X4 viruses than to R5 viruses (55–58). These findings may explain why X4 viruses are less common than dual-tropic viruses at PHI (13–15). Other studies proposed that CXCR4-using viruses are more effectively inhibited than R5 viruses by host immunity mediated by cytotoxic CD8+ T lymphocytes and NAbs (59–63). In particular, in some individuals inoculated with R5 and X4 viruses, selective repression of X4 viruses at the time of antibody seroconversion has been reported (61, 63). Although these data have contributed to the general idea that X4 viruses are more sensitive to neutralizing antibodies, they cannot be generalized. A recent study has shown that R5 T/F viruses can evolve into CXCR4-using viruses to evade the host immune system (64). In a longitudinal study, our laboratory has also demonstrated that CXCR4-using viruses can persist for years after PHI, even under effective anti-retroviral therapy (65). These data demonstrate the potential of CXCR4-using viruses to establish systemic infection in the host.

Although rare, cases of transmission of CXCR4-using viruses have been reported. As mentioned above, Δ32/Δ32 homozygous individuals in whom CCR5 is not functional could be infected by CXCR4-using viruses (66–71), which, in some cases, have been identified as dual-tropic viruses (72–75) or pure X4 viruses (76–78). Transmission of CXCR4-using viruses in individuals with wild-type CCR5 has also been reported (79–81). Dual-tropic T/F *env* sequences could also be isolated from individuals diagnosed at PHI, although much less frequently compared with R5 T/F *envs* (20, 40, 42) and not in all studies (31). To date, only one case of a strictly X4-tropic T/F virus transmitted through the mucosal route has been reported (81). Actually, when transmission pairs were studied, the presence of CXCR4-using viruses in the donor did not necessarily predict their presence in the recipient (32). Some authors have suggested that transmission of CXCR4-using viruses is associated with a threshold proportion of CXCR4-using viruses in the donor’s blood (82). This is in line with the fact that the proportion of CXCR4-using viruses in the genital compartment is generally low but increases when their viral load in the blood is high (83, 84). Whether viral load influences transmission in the same way, depending on whether viruses are R5- or X4/R5X4-tropic, is, however, not well known. It is well established, however, that the quantity of X4 viruses in people living with HIV (PLWH) is generally much lower than that of R5 viruses. As mentioned above, the proportion of PLWH harboring CXCR4-using viruses is low at PHI, but can increase dramatically (up to 50%) as the infection progresses, depending on HIV subtype, whether or not the infection is treated, or the sample from which the viruses are isolated (plasma, peripheral blood mononuclear cells [PBMCs], sexual fluids) (85–92). However, the CXCR4-using viruses that emerge in these PLWH most often coexist with R5 viruses and are even in the minority (particularly in the chronic phase) (83, 86, 91, 93). Collectively, these data led some to propose that CXCR4-using viruses are rarely transmitted primarily because their proportion in donors is low (94).

To date, however, it has not been clearly established that CXCR4-using viruses are intrinsically less transmissible than R5 viruses. Actually, it is not known whether X4/R5X4 Envs lack particular determinants required for viral transmission, which would be overrepresented in R5 T/F Envs. A comprehensive analysis of the genetic identity and functions of X4/R5X4 T/F Envs has not yet been carried out to answer these questions. In this study, we used single-genome amplification (SGA) to characterize R5 or X4/R5X4 Env populations in the plasma of 22 recently infected individuals. Our results support the idea that X4/R5X4 Envs are genotypically and phenotypically equipped for viral transmission and the establishment of infection. Mathematical modeling of Env evolution and phylogenetic analysis also revealed that X4/R5X4 viruses undergo a genetic bottleneck during transmission, as shown for R5 viruses, suggesting that barriers determining this bottleneck are common to CCR5- and CXCR4-using viruses. Neutralization assays, however, showed specific sensitivity patterns for PHI-derived X4/R5X4 Envs to sCD4 and bNAbs, compared to R5 viruses. This may impact bNAb-based passive immunization protocols, which are being considered for HIV prevention and treatment. Our results also suggest that immunogen combinations from CXCR4-using and R5 T/F Envs may represent a strategy to elicit bNAbs and broaden the breadth of their anti-HIV response.

## RESULTS

### Study population, viral tropism, and Fiebig stage

The plasma samples that were used in this study harbored HIV-1 subtypes B (20 samples) or CRF02 (4 samples) and have been classified into two categories according to whether diagnosis was made at an early (Fiebig stages I-II-III) or late (Fiebig stages IV-V-VI) stage of PHI (Table S1) (95). The tropism (i.e., coreceptor usage) of viral populations in the plasma was determined phenotypically on U87 cells using an assay described previously (15). This assay, which relies on the production of recombinant viruses (recombinant virus assay or RVA) expressing *env* populations amplified from plasma, showed strong concordance with clinically validated assays (Trofile, ESTA) and next-generation sequencing combined with genotyping tools for determining viral tropism (96–98). Infectivities of the recombinant viral populations in U87-CD4-CCR5 and U87-CD4-CXCR4 cells led us to define three groups of samples according to Fiebig stage and depending on whether they could use CCR5 only (R5_I–III_ and R5_IV–V_ groups) or CXCR4 ± CCR5 (XP group) (Table S1). All but two of the samples in group XP were isolated at Fiebig stages IV and V (XP1 was isolated earlier, and XP9 was isolated at Fiebig stage VI). Figure S1A and B shows that CD4+ T-cell counts at the time of diagnosis were similar between the three groups and that viral load (VL) was reduced in group XP, compared with group R5_I–III_. However, it is difficult to draw conclusions from this. Indeed, VL is highly dynamic during PHI, and the lower VL observed in group XP compared with group R5_I–III_ could simply reflect the fact that they were isolated at a later time point. Infection inhibition assays of primary CD4+ T lymphocytes (CD4TL) in the presence of excess maraviroc (MVC), AMD3100, or a mixture of both antagonists have made it possible to further categorize viruses in group XP according to the relative efficiency with which they use the two coreceptors: R5_X4_ (more efficient use of CCR5), _R5_X4 or X4 (more efficient or exclusive use of CXCR4), and R5X4 (equally efficient use of CCR5 and CXCR4) (Fig. S2; Table S1). CCR5 genotyping for the presence of the Δ32 mutation previously investigated in some samples, the sex, and the mode of contamination of individuals are also shown in Table S1 or its legend.

### Identification of transmitted X4/R5X4 *env* sequences

**Fig 1:**
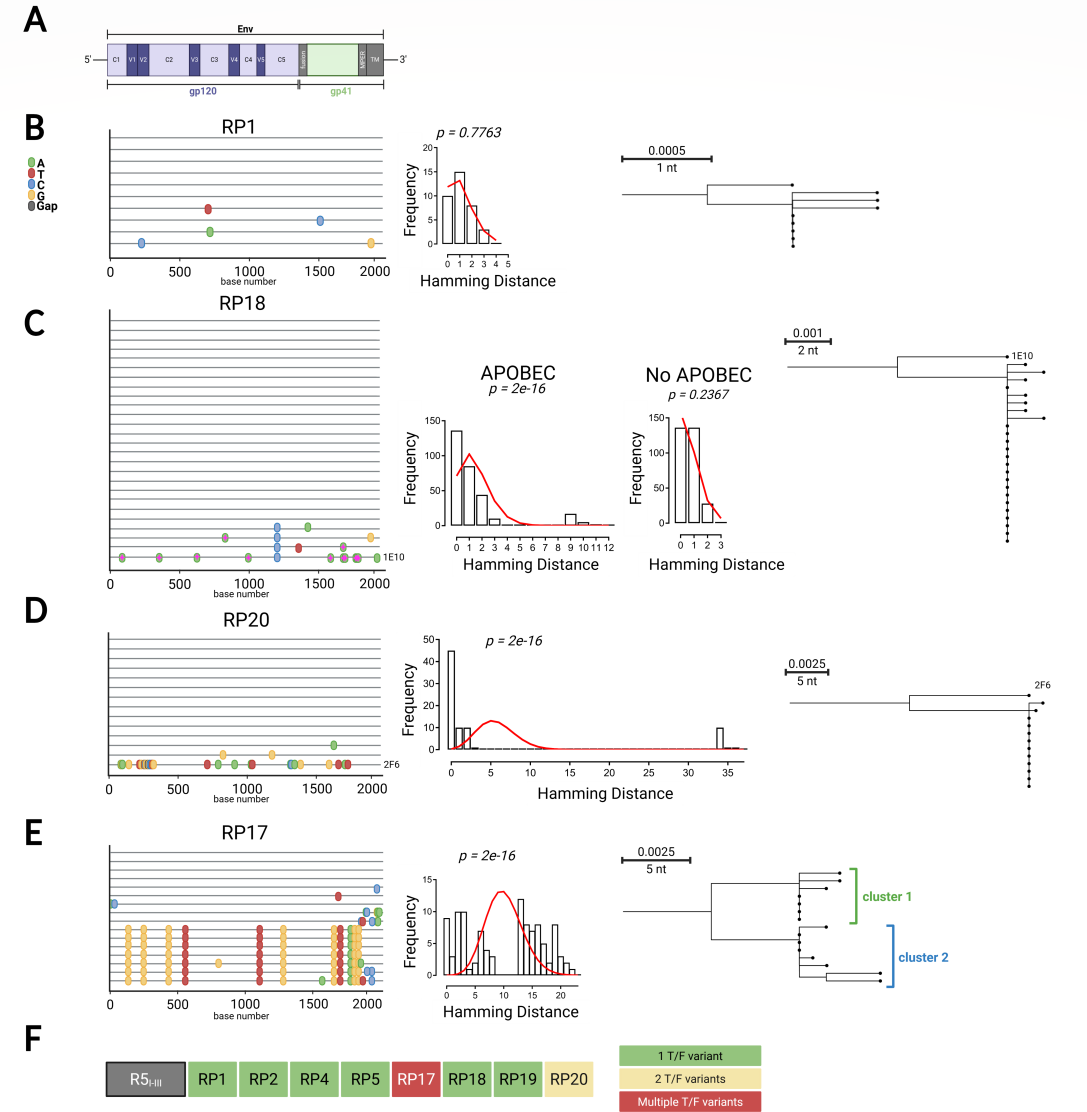
Diversity of plasma *env* populations in group R5_I–III_. (**A**) The *env* region amplified by SGA covers gp120 and the ectodomain and transmembrane regions of gp41. (**B–E**) Different diversity profiles of R5 *envs* isolated at early Fiebig stages were analyzed by (from left to right) haplotype alignment using the *Highlighter* tool, analysis of Hamming Distance (HD) frequency distributions, and maximum-likelihood phylogenetic trees. In the *Highlighter* plots, each line represents an individual sequence where the position of mutations is indicated by a colored dot in comparison with the consensus sequence (top line). In the HD frequency histograms, the red line represents the modeled results assuming transmission of a single variant and no selective pressure, as determined using the Poisson-Fitter tool. The *P*-value indicates the goodness of fit between the experimental and theoretical data. A *P*-value below 0.05 indicates that *env* diversity does not reflect homogeneous transmission and/or a mutation distribution obeying Poisson’s law. On the phylogenetic trees, each dot represents an individual *env* sequence. The scales are expressed in genetic distance and number of nucleotides (nt), calculated for a sequence length of 2,000 bp. The distribution of HD frequencies for RP1 follows a Poisson distribution (*P* > 0.05), which is consistent with an infection initiated by a single variant. RP18 contains a sequence (1E10) enriched in APOBEC mutations (represented by pink stars), leading to a deviation from the Poisson distribution (*P* < 0.05) and star-like phylogeny. Excluding these mutations from the analyses using the Poisson-Fitter tool (No APOBEC) demonstrates that *env*s in RP18 originate from a single T/F variant. In RP20, results are best explained by the transmission of two sequences, haplotype 2F6 and the consensus sequence. In RP17, two homogeneous *env* clusters are apparent. For both clusters, however, the estimated time since the MRCA was higher than expected for Fiebig stage I. This suggests that these clusters result from the transmission of more than one variant. (**F**) Number of transmitted/founder *envs* in group R5_I–III_, as detailed in the text.

We used SGA and Sanger sequencing to characterize individual *env* sequences (schematized in Fig. 1A) in plasma from individuals belonging to groups R5_I–III_ (eight samples), R5_IV–V_ (six samples), and XP (eight samples) (Table S1). Two samples in groups R5_I–III_ (RP3) and R5_IV–V_ (RP13) were not used here, as their available quantity and viral load were too low. The Geno2Pheno algorithm predicted that all Envs in the XP group can use CXCR4 as coreceptor (i.e., are X4- or dual-tropic), except in sample XP9, where a mixture of R5 and CXCR4-using Envs was present (see Fig. S3 and its legend). These results are therefore in perfect agreement with those of the phenotypic tropism assays (Table S1; Fig. S2), as previously shown (99). The median number of *env* sequences analyzed *per* plasma was 17 and was similar in the three groups (Fig. S4A). The number of different sequences (haplotypes) was, however, lower in group R5_I–III_ than in the other groups, consistent with *env* diversity increasing over time after transmission (Fig. S4B). Group XP also had fewer haplotypes than group R5_IV–V_. This suggests that CXCR4 usage is not associated with an increase in the number of transmission events or the degree of Env diversification during PHI.

To investigate this further, we analyzed the phylogenetic relationships of *env* sequences in each plasma, in particular to determine whether they originate from the transmission of one or more variants. Using the Poisson-Fitter tool previously described (100), *env* diversity in four of the eight plasmas in group R5_I–III_ (RP1, Fig. 1B and RP2, 4, and 5, Fig. S5) could be explained by a model of homogeneous transmission and random accumulation of mutations (i.e., free of selection pressure). Accordingly, the frequencies of HDs, which measure the number of nucleotides that differ between two sequences in the plasmas, followed a Poisson distribution, and all sequences coalesced in a star-like phylogeny to a single consensus sequence, the most recent common ancestor (MRCA) sequence. Additionally, the estimated times to the MRCA were consistent with the Fiebig stage (<25 days). The four remaining samples, however, showed increased mutation rates not compatible with the Poisson distribution (i.e., *P* < 0.05). In RP18, the *Highlighter* tool described in reference 100 showed that the deviation from the Poisson distribution was due to the presence of APOBEC3G/F-mediated G-to-A mutations, which were particularly enriched in haplotype 1E10 (Fig. 1C). Indeed, by excluding these mutations from the analysis using the Hypermut program from the Poisson-Fitter tool, we found that the sample actually originated from a single variant transmitted at an estimated date of 16 days (Fig. 1C). In RP20, one haplotype contributed to the HD frequency distribution not obeying the Poisson’s law (2F6) and to an overestimation of the time to the MRCA (120 days). This haplotype contained an abnormally high number of mutations, most of which were not APOBEC mutations (Fig. 1D). When analyzed separately, the other sequences in RP20 originated from a single transmitted sequence (the consensus sequence) at an estimated time of 11 days. At this very early stage, it is unlikely that the mutations in 2F6 resulted from immune selection. Thus, we concluded that the *env* sequences in RP20 most likely originated from the transmission of two sequences, 2F6 and the consensus sequence, although it remains unclear why we were unable to detect additional haplotypes from 2F6. Like RP20, RP19 also showed one sequence that diverged from the others, but to a lesser extent (Fig. S5D). Haplotype 3G7 actually contained six mutations, of which one was an APOBEC mutation, and the estimated time since the MRCA was 25 days, thus at the very end of the Fiebig stage III. Thus, it cannot be ruled out that the mutations in 3G7 may have resulted from the initiation of an immune response in the individual from whom RP19 was isolated. To get further information into the relationships between *envs* in the last sample (RP17), phylogenetic trees were constructed using the maximum likelihood method and rooted at midpoint (Fig. 1E). This approach (which was also used for the other samples) identified two distinct *env* clusters in RP17, which, when analyzed separately (after excluding the contribution of APOBEC mutations in cluster 1), appeared homogeneous and to have evolved in a star-like phylogeny (*P* = 0.52 and *P* = 0.09 for clusters 1 and 2, respectively). For both clusters, however, the time since the MRCA was disproportionately high (36 and 85 days), and thus not consistent with Fiebig stage I. This suggests that RP17 may have originated from the transmission of multiple (>2) closely related variants. Considered altogether, however, our data confirm that infection with R5 viruses most often results from transmission of a single viral variant (Fig. 1F).

**Fig 2:**
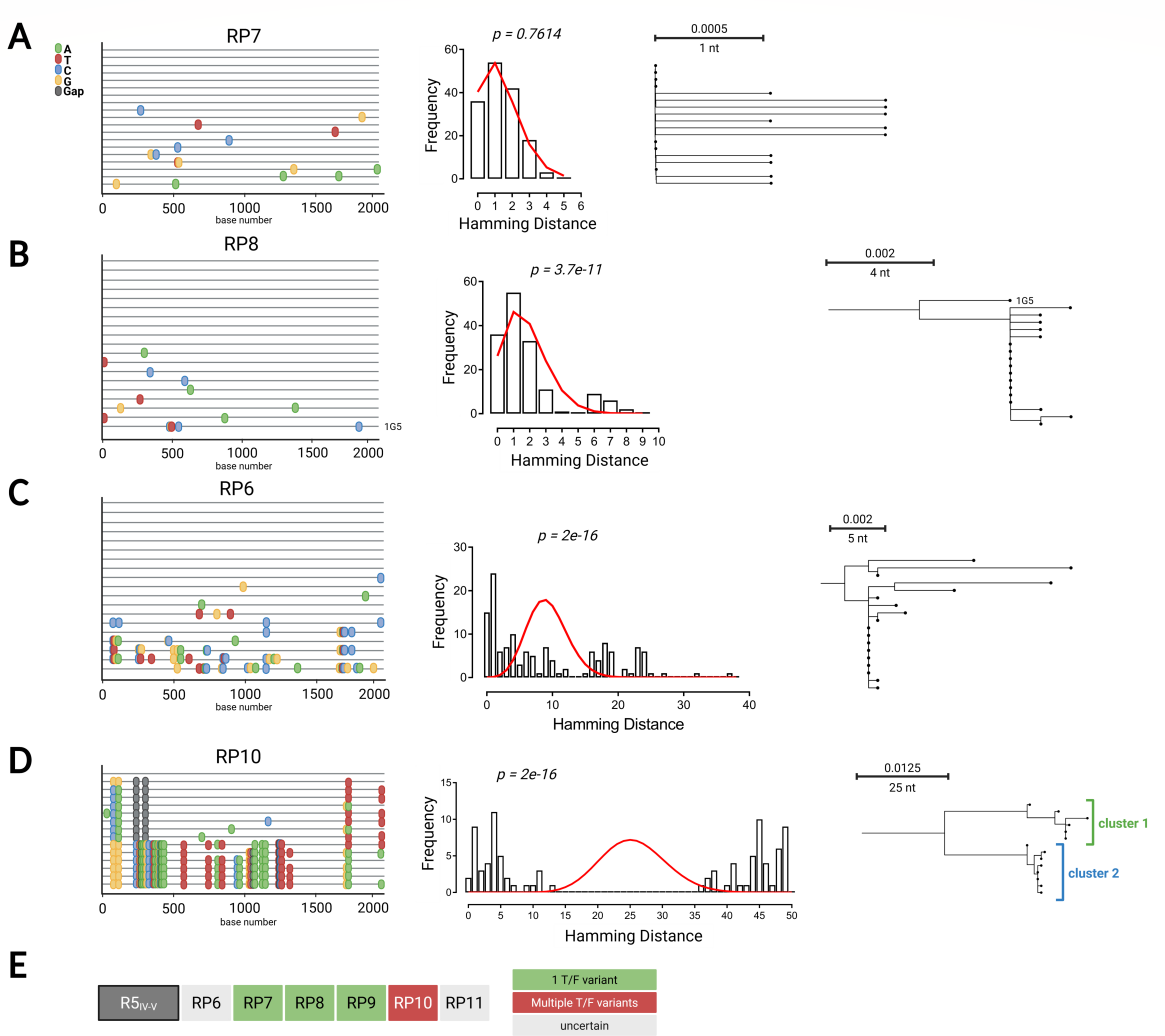
Diversity of plasma *env* populations in group R5_IV–V_. Diversity of *envs* in plasma samples RP7 (**A**), RP8 (**B**), RP6 (**C**), and RP10 (**D**) was analyzed as in Fig. 1. Data for RP7 suggest that it originated from the transmission of a single variant. In RP8, one haplotype (1G5) contributed to deviation from the Poisson distribution of mutations. In RP6, the HD frequency distribution did not conform to a Poisson distribution, and the time since MRCA was higher than expected based on the Fiebig stage (200 days). RP10 showed two *env* clusters, for which it was not possible to determine whether they originated from a single T/F sequence. (**E**) Number of transmitted/founder *envs* in group R5_IV–V_. “Uncertain” indicates that it was not possible to determine whether the increased *env* population diversity reflects transmission of multiple variants or selective pressure.

In group R5_IV–V_, the same approach showed that *env* sequences in RP7 originated from a single T/F sequence transmitted 29 days ago (Fig. 2A). Sequences in RP8 (Fig. 2B) and RP9 (Fig. S6A) have also evolved following a star-like phylogeny, and the estimated times of transmission were consistent with the Fiebig stage (30 and 46 days, respectively). However, one haplotype in each sample (1G5 in RP8 and 2D9 in RP9) contained more mutations (six in each) than expected according to the model. As discussed above for RP19, immune selection may have contributed to this pattern. In samples RP6 (Fig. 2C) and RP11 (Fig. S6B), the HD frequency distribution did not conform to a Poisson distribution, leading to overestimated times since the transmission (200 and 112 days, respectively). The analysis with *Highlighter* showed that APOBEC3G/F had contributed only marginally to the increased diversity of *env* sequences in these samples. We also investigated whether the increased diversity of these samples could be explained by recombination events between different transmitted *envs*, as shown previously (20, 24). We therefore conducted analyses using a Genetic Algorithm for Recombination Detection (GARD), the Los Alamos’ Recombinant Identification Program (RIP), and the Los Alamos’ Recombination Analysis Program (RAPR). The *env* sequences, in RP6 and RP11, but also in the other samples of this study, did not show evidence of recombination. Thus, we conclude that the high diversity of *envs* in RP6 and RP11 could have resulted from transmission of multiple closely related variants and/or reflect selection pressure from the host immune response. Finally, *env* sequences in sample RP10 segregated into two clusters (Fig. 2D). When analyzed separately, the distribution of mutations in both clusters followed a Poisson distribution but not a star-like phylogeny. Additionally, the estimated time to MRCA in cluster 1 was a bit higher than expected based on Fiebig stage IV–V (142 days). However, it was not possible to determine whether RP10 results from the transmission of two variants that diversified in response to immune pressure, or from transmission of more than two variants (Fig. 2E).

**Fig 3:**
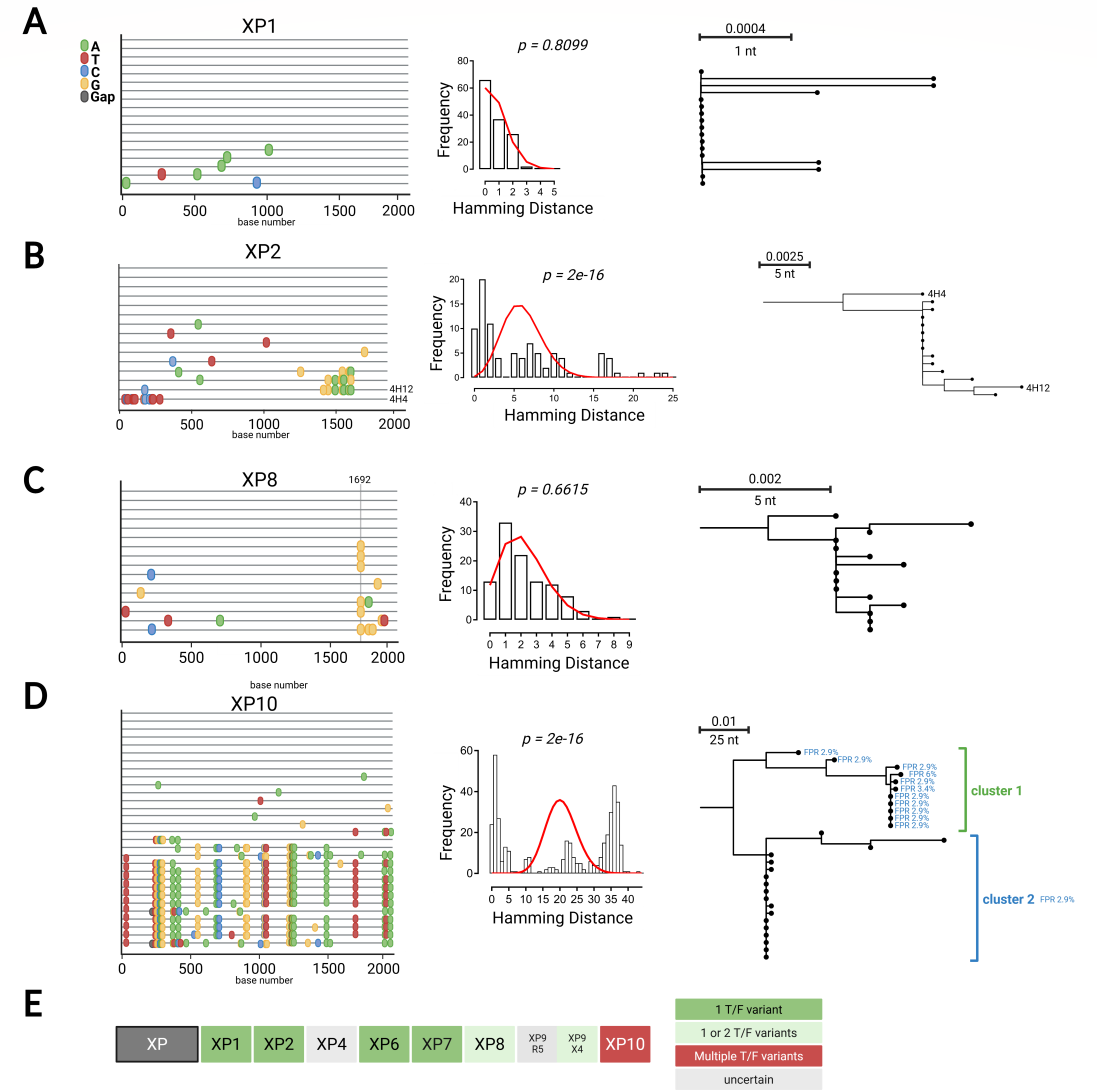
Infection by CXCR4-using viruses is most often initiated by one or two related *env* sequences. (**A–D**) Diversity of *envs* in plasma samples XP1 (**A**), XP2 (**B**), XP8 (**C**), and XP10 (**D**) was analyzed as in Fig. 1 (for details, see the text). Data for XP1 and XP2 are consistent with the transmission of a single T/F sequence. XP8 may have originated from two variants with a T or G at position 1692. Sample XP10 showed two genetically distant *env* clusters, which were themselves highly heterogeneous. (**E**) Number of transmitted/founder *envs* in group XP. The term “uncertain” for samples XP4 and XP9_R5_ indicates that it was not possible to determine whether the increased *env* population diversity reflects transmission of multiple variants or selective pressure. FPR scores for the different *envs* in XP10 were determined using G2P.

The only sample from Fiebig stage II–III in group XP (XP1) and sample XP6 (Fiebig stage IV–V) contained identical or nearly identical *env* sequences that coalesced to a single T/F sequence (Fig. 3A; Fig. S7B). In XP7 (Fig. S7C), two haplotypes (3C2 and 3A7) were abnormally enriched in mutations. However, they were clearly derived from other sequences in the sample, as indicated by shared mutations with other haplotypes. This confirms that XP7 also originated from the transmission of a single variant. Some haplotypes in XP2 also showed more mutations than predicted by the model, including those exhibiting an APOBEC signature (Fig. 3B). We analyzed the distributions of HD frequencies while excluding haplotype 4H4 and APOBEC mutations and found that they followed a Poisson distribution (*P* = 0.13). The estimated time to the MRCA was also consistent with Fiebig stage V (71 days). Haplotype 4H4 shared a mutation with haplotype 4H12, indicating that these two haplotypes are linked, and thus that haplotype 4H4 was not co-transmitted with the consensus sequence. This is consistent with the *envs* in XP2 originating from a single transmitted sequence. In XP8, a Poisson distribution of HD frequencies could be observed (Fig. 3C), and the estimated time since transmission was consistent with Fiebig stage V (47 days). However, the sample was not described by a star-like phylogeny. Actually, the *Highlighter* plot suggested the presence of two distinct sub-lineages in XP8, depending on whether a T or G is present at position 1692. When analyzed separately, these sub-lineages showed a star-like phylogeny and coalesced to a single sequence at a time consistent with Fiebig stage V. These data suggest that XP8 may have originated from two variants, although we cannot exclude the possibility that the two sub-lineages resulted from *env* diversification occurring after transmission. The other three samples (XP4 and XP9 in Fig. S7 and XP10 in Fig. 3D) in group XP were clearly more heterogeneous, suggesting that they may have evolved from transmission of multiple variants and/or reflect the fact that they were isolated later than the other samples in the group. Analyses with RIP, GARD, and RAPR ruled out the possibility that this increased diversity arose from recombination events. APOBEC mutations also contributed only marginally to the increased diversity. XP10 showed two genetically distant *env* clusters, which were themselves highly heterogeneous (i.e., within which mutations did not follow Poisson distribution and star-phylogeny) (Fig. 3D). XP9 also originated from multiple variants, which could be subdivided into two clusters corresponding to the CXCR4-using (cluster 1) and R5 (cluster 2) *env* sequences that coexist in this sample (Fig. S7D). Individual analysis using the Poisson-Fitter tool indicated that cluster 1 may have originated from one or two variants (like XP8). In contrast, R5 *env* sequences were heterogeneous and not consistent with a Poisson distribution of mutations, and their MRCA could not be determined. Therefore, the fact that these two clusters originated from different transmission events cannot be ruled out.

Altogether, these data indicate that infection by CXCR4-using viruses is most often established by a limited number of virus variants (Fig. 3E). Four of the eight samples in group XP originated from the transmission of a single variant. For two of them (including XP9_X4_), it was not possible to determine whether they resulted from one or two variants. Only one (XP10) was clearly shown to derive from more than two variants, while for the remaining sample (XP4), we were not able to determine whether its increased sequence heterogeneity was due to the transmission of multiple variants or to immune selective pressure. Therefore, these results are highly similar to those observed for the samples of group R5_IV–V_ isolated at the same Fiebig stage, indicating that CXCR4-using viruses undergo a genetic bottleneck during transmission, as shown for R5 viruses. Of note, tropism analysis with Geno2Pheno indicated that the T/F variants in samples XP can use CXCR4, ruling out that *env* sequences in group XP evolved from R5 T/F viruses.

### Complexity of CXCR4-using *env* sequence populations at PHI

**Fig 4:**
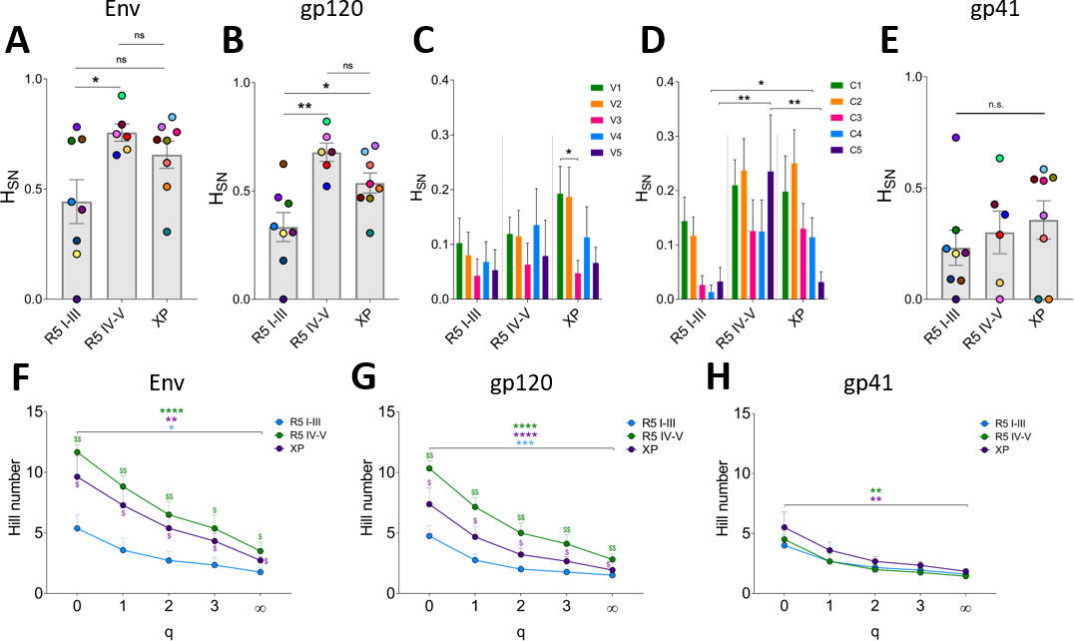
Complexity of CXCR4-using plasma *env* populations at PHI. (**A–E**) Normalized Shannon entropy H_SN_ for *env* populations in the plasma samples of groups R5_I–III_, R5_IV–V_, and XP was calculated considering full-length *env* (as indicated in Fig. 1A) (**A**), gp120 (**B**), variable loops (**C**), constant regions (**D**), or gp41 (**E**). Each colored dot represents a given sample as detailed in Fig. S4C. Means ± SEM are shown. Statistics: Mann–Whitney *U*-test. (**F–H**) Hill numbers of order *q* = 0 to ∞ for plasma *env* populations in groups R5_I–III_, R5_IV–V_, and XP were calculated considering full-length *env* (**F**) or the gp120 (**G**) or gp41 regions (**H**). Each data point represents the mean Hill numbers (± SEM) in each *env* group. For each *env* group, the evolution of Hill number values as a function of q was analyzed using a one-way ANOVA followed by Tukey’s test. *, **, ***, and **** indicate *P* < 0.05, *P* < 0.01, *P* < 0.001, and *P* < 0.0001, respectively. Statistical differences in the Hill numbers for groups R5_IV–V_ and XP were assessed in comparison to group R5_I–III_ using the Mann–Whitney *U*-test. $ and $$ indicate *P* < 0.05 and *P* < 0.01, respectively.

We sought to further quantify the complexity of *env* populations in the three groups, using classical indices used to describe viral quasispecies dynamics, taking into account both the frequency and diversity of haplotypes (101), and to determine which regions of *envs* were involved. The nucleotide diversity (π_e_), which reflects haplotype diversity, was of the same order in groups R5_I–III_, R5_IV–V_, and XP, and was generally low, except for samples derived from the transmission of multiple variants (RP10, XP9, and XP10) (Fig. S8). Therefore, to further describe the complexity of *env* populations in the three groups, and determine whether and how it varies with viral tropism (i.e., coreceptor usage) and over time during PHI, we calculated their normalized Shannon entropy (H_SN_). H_SN_ expresses the probability that a randomly selected *env* sequence represents a given haplotype in the whole *env* population. This probability decreases as sample complexity increases between H_SN_ values of 0 and 1. Groups R5_IV–V_ and XP had higher H_SN_ values than group R5_I–III_ (Fig. 4A), reflecting an increase in *env* population complexity during PHI. This effect was restrained to gp120 (Fig. 4B), most often in V1 and V2 loops, but also in constant regions (Fig. 4C and D). The H_SN_ values for the constant regions were higher in groups R5_IV–V_ and XP than in group R5_I–III_, although this reached statistical significance only for C4 (XP) and C5 (R5_IV–V_). Only H_SN_ of C5 in group XP was found not to vary, compared to group R5_I–III_, perhaps reflecting a peculiarity in CXCR4-using Envs. When considering only the gp41 subunit, H_SN_ values were not significantly different between the three groups (Fig. 4E).

We then calculated Hill numbers of order *q* to characterize how the complexity of *env* populations varies with haplotype abundance (Fig. 4F). The contribution of minority haplotypes to complexity decreases as *q* increases, and for *q* = ∞, only majority haplotypes matter (101). For all three *env* groups, considering only the gp120 region, Hill numbers decreased significantly with increasing *q* (Fig. 4G). This indicates a major contribution of minority haplotypes to complexity, albeit less pronounced in group R5_I–III_. Groups R5_IV–V_ and XP showed similar Hill numbers, which were significantly higher than those of group R5_I–III_, indicating greater complexity. These differences persisted at *q* = ∞. This indicates that the majority haplotypes also contribute to differences in *env* population complexity between the three groups, although less than the minority haplotypes. When considering the gp41 region, Hill numbers were comparatively lower, not different between the three groups, and less sensitive to increasing *q* (Fig. 4H). This confirms that gp41 is less subject to variation during PHI than gp120.

Overall, these data therefore show that the complexity of *env* populations increases during PHI, in a way that impacts the sequence of gp120 but not gp41, and independently of viral tropism. Of note, there were no differences in the relative proportions of nonsynonymous (dN) and synonymous (dS) mutations in *env* sequences between groups R5_IV–V_ and XP (see Fig. S9). This suggests that the complexity of Env protein populations also varies similarly, regardless of whether they are R5- or X4/R5X4-tropic.

### Potential *N*-linked glycosylation sites and loop length in CXCR4-using Envs at PHI

**Fig 5:**
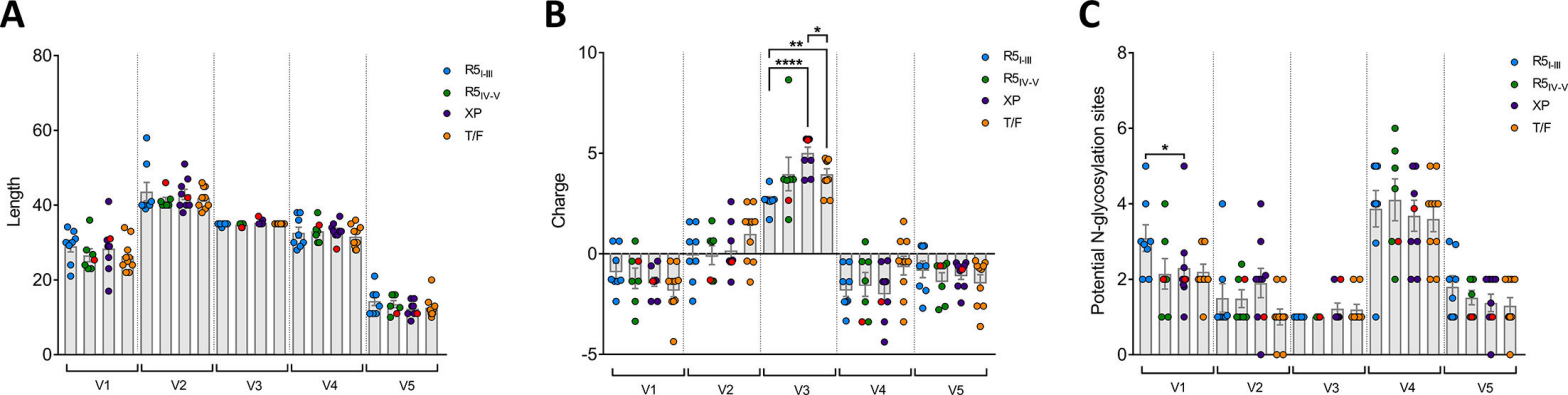
CXCR4-using and R5 Envs share common structural traits associated with viral transmission. (**A and B**) Amino acid length and net charge, respectively, of the variable loops of gp120 in Envs of groups R5_I–III_, R5_IV–V_, and XP. (**C**) Number of potential N-linked glycosylation sites in these variable loops. For comparison purposes, data are also shown for the R5 T/F Envs pWITO.c/2474, pCH040.c/2625, pCH058.c/2960, pCH077.t/2627, pCH106.c/2633, pRHPA.c/2635, pTHRO.c/2626, pREJO.c/2864, pTRJO.c/2851, and pSUMA.c/2821. Each data point in the different groups refers to a given plasma sample. For XP9, clusters R5 and X4 are represented by red dots within their respective groups (R5_IV–V_ and XP). Within each group, results represent the mean (A and C) or median (B) values calculated from all samples. Error bars represent SEM. Statistics: Mann–Whitney *U*-test: *: *P* < 0.05, **: *P* < 0.01, ****: *P* < 0.0001.

*N*-linked glycosylations and loop length in HIV-1 Env have been proposed to influence virus transmission and initiation of infection. These characteristics were thus compared between groups R5_I–III_, R5_IV–V_, and XP (Fig. 5). Indeed, we anticipated that whether CXCR4-using Envs differ from R5 Envs in their number of potential *N*-linked glycosylation sites (PNGS) or loop size, this could explain why they are less frequently transmitted. Loop length varied greatly between haplotypes within each group, with the exception of V3, whose size (here 35 amino acids) is known to remain constant (Fig. 5A). Overall, however, average loop length was similar between groups and consistent with previously reported R5 T/F Envs (20). There was also no difference in the net charge of V1, V2, V4, and V5 loops between the different Env types (Fig. 5B). The positive net charge for V3 was higher in the XP group than in the other groups with R5 Envs. This was partly due to the presence of a basic residue (R/K) at positions 11 and/or 25 (Table S2A through E), which is characteristic of X4 Envs (102). The number of PNGS was also highly variable in V1, V2, V4, and V5 loops, much less so in V3, as expected (Fig. 5C; Table S2). But as with their length, the number of PNGS in the loops did not vary between the different Env groups. The regions in which these PNGS are present in the loops were also highly preserved (Fig. S10; Table S2). Interestingly, all Envs in group XP contained the conserved PNGS at position 301 in V3, whereas X4 Envs that evolve from R5 T/F Envs generally lack it (64). Moreover, all XP Envs shared with R5_I–III_ and R5_IV–V_ Envs the lack of a PNGS at position 413 (Table S2), similarly as in R5 T/F Envs (35). Actually, this PNGS has been more frequently observed in chronic Envs and has been associated with decreased sensitivity to neutralization by mAb b12, which targets the CD4-binding site (35). Collectively, these data therefore show that CXCR4-using and R5 T/F Envs share common structural traits associated with viral transmission.

### Cellular tropism of CXCR4-using Envs at PHI

**Fig 6:**
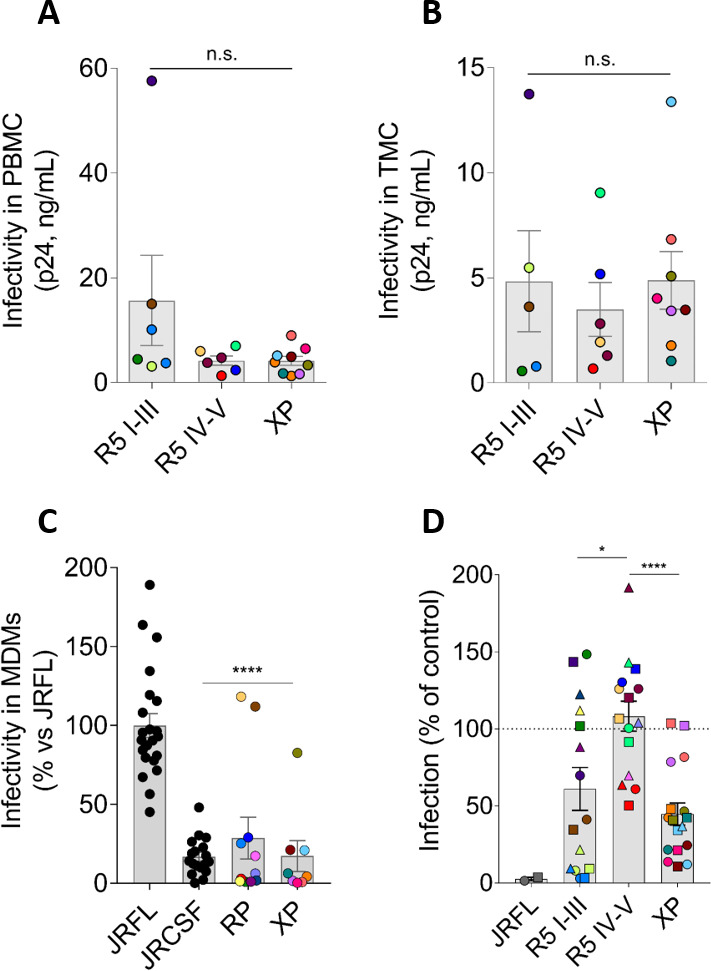
Influence of viral tropism and PHI stage on the cellular tropism and sensitivity to sCD4 neutralization of HIV-1 Envs. (**A and B**) Infectivity of recombinant viruses pseudotyped with plasma Env populations from groups R5_I–III_, R5_IV–V_, or XP was assessed in PBMCs (**A**) or TMCs (**B**). Cells were inoculated with equal amounts of viruses (10 ng of p24) and then further incubated in the culture medium for 4 days at 37°C. Each data point represents the mean infectivity (in ng/mL of Gag p24 in the cell supernatant) of Envs from a given plasma sample (color coded as indicated in Fig. S4C), determined from at least three independent experiments on cells from distinct donors and with two independent virus preparations. Results represent mean infectivity values (± SEM) of the different samples. (**C**) Infectivity in MDMs of Luc2-expressing recombinant viruses pseudotyped with CXCR4- or CCR5-using Env populations, or the M-tropic JR-FL or non-M-tropic JR-CSF Envs used as controls. MDMs were infected with constant doses of viruses (equivalent to 50,000 RLU in CD4+ T cells). Infectivities (in RLU) were determined 48 h post-infection by measuring the luciferase activity in the cell lysates. Each point represents the mean infectivity of a sample measured at least three times on cells from different donors. Results are expressed as percentages of JR-FL infectivity (100%) and represent means ± SEM of the different infectivity values measured in each group. Data points for JR-FL and JR-CSF represent technical replicates from four independent experiments. (**D**) Soluble CD4 neutralization of recombinant viruses pseudotyped with plasma Env populations of groups R5_I–III_, R5_IV–V_, or XP or JR-FL Env. Constant doses of viruses (3 × 10^6^ RLU) were incubated with 300 nM sCD4 for 1 h at 37°C, before being added to TZM-bl cells. Luciferase activity was measured 48 h post-inoculation, and results were expressed in percentages of infection compared to the control condition in the absence of sCD4. Each color represents the mean residual infectivity of a given virus population measured at least twice, and each symbol represents an independent virus production. Statistics in the panels were run using the Mann–Whitney *U*-test: *, *P* < 0.05; ****, *P* < 0.0001; n.s., non-significant.

Lower infectivity in cells at sites of transmission and early replication (e.g*.*, lymphoid tissue) could contribute to CXCR4-using viruses initiating new infections less frequently than R5 viruses. To address this point, we produced recombinant virus populations expressing Envs from the plasmas of individuals in groups R5_I–III_, R5_IV–V_, and XP and then measured their capacity to infect human PBMCs and tonsil mononuclear cells (TMCs), a model of secondary lymphoid organ. PBMCs or TMCs were inoculated with identical doses of viruses, and the Gag p24 level in the supernatants was measured. No difference was observed between the different viruses, indicating similar infectivity in T cells (Fig. 6A and B). We then assessed their capacity to infect monocyte-derived macrophages (MDMs), following the approach we recently reported (103). Viruses pseudotyped with Envs from the M-tropic and non-M-tropic HIV-1 strains JR-FL and JR-CSF, respectively, were used as internal controls. Compared to JR-FL, all virus populations, regardless of their viral tropism, displayed strongly reduced infectivity in MDMs, similarly to JR-CSF (Fig. 6C). These results thus extend to CXCR4-using viruses previous findings on R5 viruses showing that viruses in acute infection are T-tropic but little or not M-tropic (21, 42). They also suggest that, similarly to T/F R5 viruses, CXCR4-using viruses are limited in their ability to bind CD4, a characteristic shared by non-M-tropic viruses (43). To address this point, we measured the sensitivity of viruses pseudotyped with Envs R5_I–III_, R5_IV–V_, or XP to neutralization by sCD4 in TZM-bl cell infection experiments, as previously described (104, 105). We used sCD4 at a concentration of 300 nM, that is, a concentration at which the M-tropic strain JR-FL was fully inhibited (Fig. 6D). In contrast, all viruses carrying the other Envs were only partially or not inhibited with sCD4, suggesting that they adopt a conformation where the CD4-binding site is less exposed, compared to JR-FL. Interestingly, compared with R5 Envs isolated at the same Fiebig stage IV-V, XP Envs were significantly more sensitive to sCD4. This effect was most pronounced for X4_R5_ viruses, that is, viruses using CXCR4 more efficiently than CCR5 (XP7, XP9, XP10, and XP6) (see Fig. S2). Envs in group R5_I–III_ were also more sensitive to sCD4 than Envs in group R5_IV–V_ (Fig. 6D). Therefore, these results suggest that Env proteins evolve in their sensitivity to sCD4 during PHI but do so differently depending on their viral tropism.

### Viral tropism influences HIV-1 sensitivity to bNAbs during PHI

**Fig 7:**
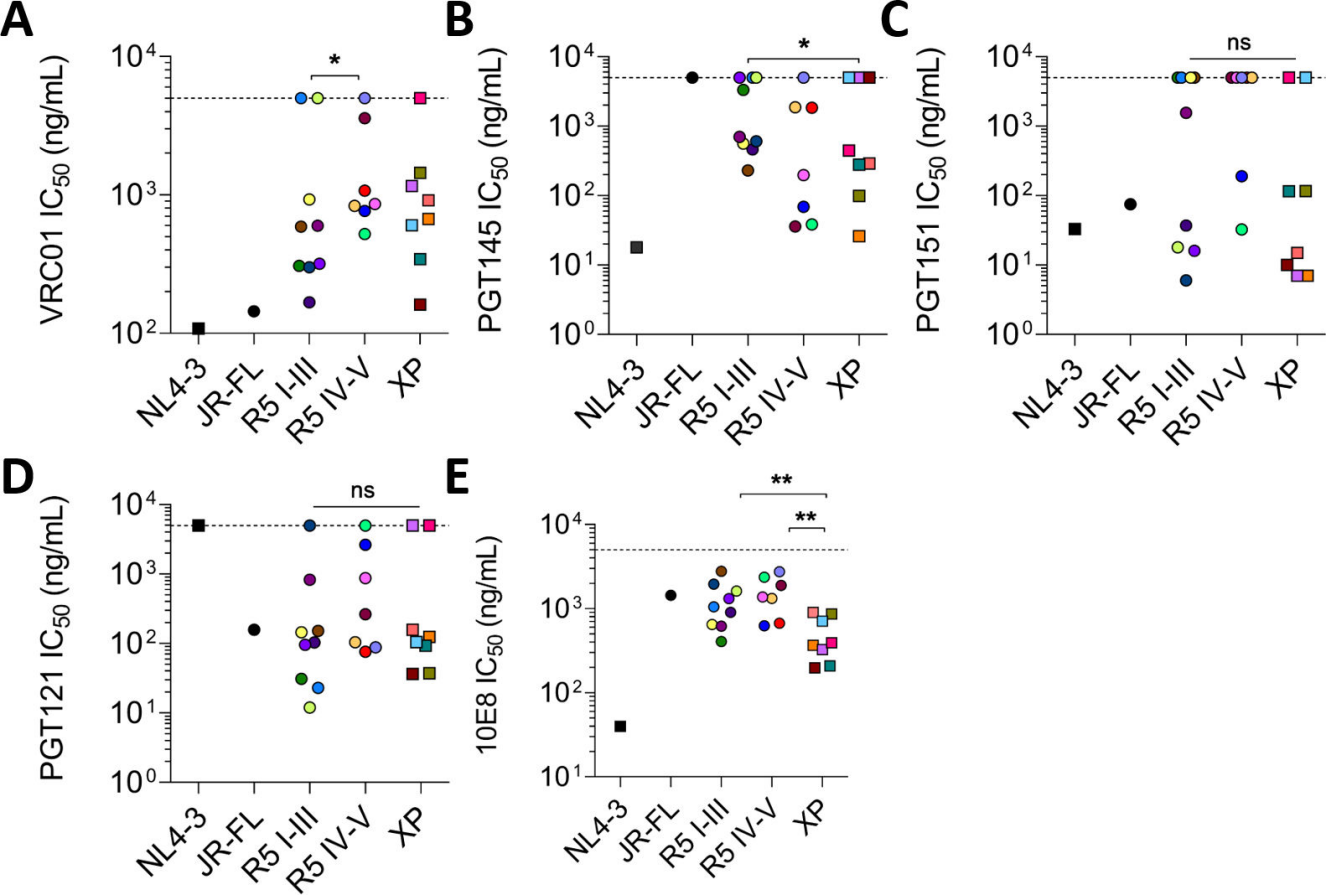
Viral tropism and PHI stage influence sensitivity of HIV-1 Envs to neutralization by broadly neutralizing antibodies. (**A–E**) Recombinant viruses pseudotyped with plasma Env populations of groups R5_I–III_, R5_IV–V_, or XP or NL4-3 or JR-FL Envs were exposed to different concentrations of bNAbs VRC01 (**A**), PGT145 (**B**), PGT151 (**C**), PGT121 (**D**), or 10E8 (**E**) and then incubated with TZM-bl cells. For each virus/bNAb pair, the mean IC_50_ values from at least three independent experiments are shown. The dashed line represents the highest antibody concentration used (5,000 ng/mL). A data point on the dashed line indicates that IC_50_ > 5,000 ng/mL. Statistics: Mann–Whitney *U*-test: *, *P* < 0.05; **, *P* < 0.01; n.s., non-significant.

The above results suggested that in the early stages of PHI, R5 and X4/R5X4 Env trimers adopt different conformations and thereby expose the CD4-binding site differently. To explore this hypothesis, viruses pseudotyped with NL4-3, JR-FL, R5_I–III_, R5_IV–V_, or XP Envs were assayed for their neutralization sensitivity to bNAbs targeting five major neutralizing epitopes in the Env trimer: the CD4-binding site (bNab VRC01), the V1/V2 apex (PGT145), the V3-glycan region (PGT121), the gp120/gp41 interface (PGT151), and the gp41 MPER domain (10E8) (Fig. 7). Viruses were incubated with varying bNAb concentrations and then added on TZM-bl cells, as described previously (104). Certain bNAbs were not active against some viruses from each group (Fig. 7). Similar observations have been reported with T/F viruses (44), suggesting that the resistance to neutralization observed here is not a property acquired during PHI (but more likely inherited from transmission). 10E8 was the only bNAb for which no resistance was observed, in agreement with the notion that MPER-targeted bNAbs are among the bNAbs with the highest neutralization breadth (106). Among Envs sensitive to VRC01, those in group R5_I-III_ were significantly more potently inhibited than those in group R5_IV–V_ (Fig. 7A). A trend toward increased sensitivity to VRC01 was also observed for some, but not all, XP group Envs. Overall, these results mimic those of neutralization with sCD4 (Fig. 6D), further confirming that Envs in groups R5_I–III_ and XP have a CD4-binding site that is generally more exposed than those in group R5_IV–V_. Conformational changes in the Env trimer apex during PHI were evidenced by our data showing that Fiebig stage IV–V Envs are more sensitive to PGT145 than the earlier Envs of group R5_I–III_, irrespective of viral tropism, although this only reaches statistical significance for group XP (Fig. 7B). This may be linked to the greater increase in V2 loop complexity in XP Envs during PHI (Fig. 4C). PGT145 did not neutralize JR-FL Env-pseudotyped viruses, in accordance with the fact that PGT145 binds weakly to JR-FL (Fig. 7B) (107).

No difference in the neutralizing activity of PGT151 and PGT121 was observed between the different Env groups (Fig. 7C and D). Of note, PGT121 was inactive against NL4-3, as previously reported (108). Finally, Envs in group XP were more sensitive to 10E8 than the other two R5 Env groups. Similarly to XP Envs, NL4-3 (X4) was very potently inhibited by 10E8, while the IC_50_ for JR-FL inhibition was of the same order as that of R5_I–III_ and R5_IV–V_ Envs (Fig. 7E). Thus, our results suggest that the ability of XP Envs to use CXCR4 is associated with their increased neutralization sensitivity to 10E8, even though most of these viruses are dual-tropic and also retain the ability to use CCR5. These data indicate that, in addition to varying with Fiebig stage, antigenicity of HIV-1 Envs is also influenced by viral tropism during PHI.

## DISCUSSION

Transmission of CXCR4-using HIV-1 strains is much less frequent than that of R5 strains (49, 94). Molecules that inhibit virus binding to CXCR4 in transmission sites and fluids (e.g*.,* CXCL12, defensins, spermine/spermidine) likely limit the transmission of pure X4 viruses (51–54), although not absolutely (76, 81). Recently, we showed that CXCL12-escape X4 isolates are common in the late stages of infection, illustrating the role of the chemokine as a selective pressure on X4 viruses (104). But transmission of dual-tropic viruses that can also use CCR5 in addition to CXCR4 is also infrequent (20, 72), which is more difficult to explain. One hypothesis is that CXCR4-using Envs, unlike R5 Envs, lack the molecular determinants required for transmission and initiation of infection. However, perhaps due to the low frequency of X4/R5X4 viruses at PHI, this issue has not yet been addressed in detail. Nevertheless, it could potentially improve our understanding of the mechanisms of HIV transmission and how to prevent it. In this study, we have characterized individual sequences of CXCR4-using or R5 Envs derived from the plasma of recently infected individuals. Envs that use CXCR4, as determined genotypically and phenotypically on U87 cells and primary CD4TL, are very similar to R5 Envs in terms of their loop length and the number and position of PNGS, two important characteristics for viral transmission and sensitivity to antibody neutralization in the early stages of infection (26, 31, 33). In particular, both types of Envs shared signatures identified in R5 T/F Envs, such as the absence of a PNGS at the end of the V4 loop, in a region adjacent to neutralizing antibody epitopes and receptor-binding regulation sites (35). R5 and CXCR4-using Envs also exhibited similar infectivities in PBMCs but also in mononuclear cells from tonsils, a SLO model. This is important as shortly after transmission, transmitted viruses target secondary lymphoid tissues and organs for intense replication and early viral reservoir formation (109). We did not aim to determine the nature of the CD4TL subpopulations targeted by R5 and X4/R5X4 viruses in this study, but recent work suggested that they might differ, influencing infection pathogenicity (81) and possibly latency. As previously shown for R5 T/F viruses (21, 42), our results also indicate that CXCR4-using Envs in recently infected individuals are non-M-tropic, consistent with their greater resistance to sCD4 compared to the M-tropic strain JR-FL. It should be noted that our study focused only on the cell-free mode of infection, whereas it has been shown by us and others that macrophages can be infected by R5 T/F viruses through viral transfer from CD4TL (110, 111). Whether the same occurs with CXCR4-using T/F viruses remains to be determined.

During PHI, our results show that mutations occur in *env* sequences within specific regions predominantly located in gp120 rather than gp41, particularly in the V1/V2 loops as well as the constant regions C1, C2, C3, and C4. Mutations were also observed in C5 for R5_IV–V_ Envs, but not for CXCR4-using Envs. However, the significance of this remains unknown. The accumulation of mutations led to an increase in Env species complexity in a manner independent of viral tropism. One limitation of our study, which prevented us from exploring this point further, is that many of our samples were collected at a stage of PHI when the pressure exerted by the host immune system was likely not yet effective. This is evidenced by the fact that many of the mutations had accumulated according to a Poisson distribution. Future analyses using later-stage samples (Fiebig stage VI) should allow investigating whether the immune response differentially affects the complexity of R5 and X4 Env populations, thereby providing insight into the capacity of these viruses to sustain infection. Actually, V1/V2 is a region of Env where HIV-1 immune escape mutations are frequent (33, 34). Here, we also observed that mutations in V1/V2 are more frequent at PHI in the absence of an immune response, and this is true for both R5 and X4 Envs. We anticipate that, similarly, preferential mutations in V1/V2 loops in response to the immune system later in infection might not be dependent on viral tropism but be related to their more exposed localization at the Env trimer apex.

Our work, therefore, does not support the hypothesis that viruses using CXCR4 as a coreceptor are not equipped for transmission. Moreover, some of the dual-tropic Envs studied here showed only minimal CCR5 use (XP7, XP9, and XP10), suggesting that their transmission did not occur through CCR5 alone (even though it can be considered that this probability is increased in transmission mucosa, where CD4TL are often more activated than those in lymphoid organs and blood, and therefore express higher levels of CCR5). Actually, the hypothesis that CXCR4-using viruses, but not R5 viruses, are counter-selected at the time of transmission would require, for definitive validation, evidence that under conditions where both types of viruses are present in equal proportions in a donor, R5 viruses are more frequently transmitted to the recipient than X4 viruses, which is, in fact, rarely the case. Even in individuals in whom CXCR4-using viruses have emerged, which is not the general case among HIV-1 subtypes, their proportion is generally low compared to R5 viruses (83, 86, 91, 93). Moreover, studies have shown that the transmission of CXCR4-using viruses is linked to a proportion threshold in the blood (82) and probably the genital tract (83, 84). In this context, although CXCR4-using viruses are likely equipped for transmission, we believe they are infrequently transmitted largely because their proportion in donors is generally low, in line with a previously proposed hypothesis (94). Interestingly, when CXCR4-using viruses are transmitted, our work shows that this occurs through a genetic bottleneck, similar to what has been observed for R5 viruses (20, 21). In six out of the eight plasmas analyzed, CXCR4-using viral species most often originated from a single (four cases) or possibly two closely related (two cases) transmitted variants. For the remaining two samples, we could not determine whether the increased diversity of the Env populations was due to the transmission of multiple variants or to immune pressure. Hence, these data suggest that the genetic bottleneck during transmission is largely independent of viral tropism, raising the possibility that the same factors may be involved in this bottleneck for both CCR5- and/or CXCR4-using viruses. These factors could lead to the selection of viruses (possibly partly stochastically) in the genital tracts or transmission fluids of either the donor or the recipient (50, 112). They could include the mucosa of the genital tracts, whose key role as a barrier to viral transmission is well established. Indeed, diseases that compromise mucosal integrity lead to an increased risk of infection acquisition during sexual intercourse and a higher number of transmitted variants (22, 113). In our study, however, among the six samples with CXCR4-using Envs where a bottleneck was observed, five resulted from sexual transmission and one from parenteral transmission (Table S1). Similarly to R5 viruses (23, 114), this suggests that the genetic bottleneck of CXCR4-using viruses may also be independent of the mode of transmission.

The present study also shows that Env antigenicity varies during PHI and differs between R5 and CXCR4-using viruses. Our data here suggest that this might be related to the accumulation of mutations from the initial stages of PHI in regions that regulate the conformation of the Env trimer, such as the V1/V2 loops, in a viral tropism-dependent manner. A study identified a pure X4-tropic T/F virus with resistance to bNAbs targeting the V3 and V1/V2 regions (81). We did not observe such results, which may reflect heterogeneity in the conformation of transmitted CXCR4-using Envs, as recently reported for R5 T/F Envs (44), or simply the fact that Envs in our study are dual- and not pure X4-tropic. In line with this latter assumption, another study showed that pure X4 Envs in PLWH tend to be less sensitive to neutralization by V3- or V1/V2-targeting bNAbs, but this was not reproduced with dual-tropic Envs (115). Overall, CXCR4-using Envs are more sensitive to sCD4 and VRC01 than the R5 Envs from the same Fiebig stage, suggesting that they adopt a more open conformation exposing the CD4-binding site more readily. Recent work showed that R5 T/F Envs can adopt partially open conformations, with the degree of opening varying from one Env to another (44). Our results, therefore, suggest that the opening of the Env trimer may vary differently in CXCR4-using T/F Envs. Another characteristic of CXCR4-using viruses from the PHI that we show here is their increased sensitivity to the MPER-targeting bNAb 10E8 compared to R5 viruses. Exposure of MPER to antibodies is induced upon Env binding to receptors and persists up to the latest stages of fusion between viral and cell membranes (116, 117). It is therefore likely that the degree of MPER exposure is closely dependent on the kinetics of viral entry, which may be slower in the case of CXCR4-using viruses, explaining their increased sensitivity to 10E8.

HIV-1 bNAbs are increasingly being considered in passive immunization protocols for the prevention, treatment, and cure of HIV infection (118). However, Env variability remains a major hurdle to the implementation of these approaches, and our results show that, in this context, Env tropism during the earliest stages of infection is a factor likely to regulate the neutralizing activity of bNAbs. The induction of bNAbs is also considered in the context of HIV vaccine development (8). In this regard, it is thought that the characteristics of T/F Env populations in the very early stages of infection drive the subsequent development of bNAbs (119). Our results, therefore, suggest that CXCR4- and CCR5-using T/F Envs, which exhibit conformational and antigenic differences, might differ in their capacity to induce bNAbs, as well as in the nature of the bNAbs they elicit. In this context, using immunogen combinations designed from CXCR4-using and R5 T/F Envs could represent an approach to increase the chances of inducing bNAbs and to broaden the breadth of their anti-HIV response.

## MATERIALS AND METHODS

### Single-genome amplification

The protocol was adapted from that of Keele et al. (20). Viral RNA (vRNA) was extracted from plasma samples (140 μL) using the QIAamp Viral RNA Kit (QIAGEN), according to the manufacturer’s instructions. Reverse transcription (RT) of an RNA amount corresponding to 20,000 copies into single-stranded cDNA was then performed using SuperScript III Reverse Transcriptase (Invitrogen). For each RT reaction, 1 µL of the antisense primer OFM19 (stock at 10 µM, 5′-GCACTCAAGGCAAGCTTTATTGAGGCTTA-3′), 1 µL of a mixture of dNTPs (at 10 mM each), and vRNA were mixed in a 15 µL final volume, heated at 65°C for 5 min, and then cooled on ice for at least 1 min. The following components were then added: 1 µL of DTT (0.1 M), 1 µL of RNaseOUT (RNase inhibitor) (40 units/µL), 1 µL of SuperScript III (200 units/µL), and 4 µL of 5× First-Strand Buffer. The reaction mix was incubated at 50°C for 1 h, then at 55°C for 1 h, heat-inactivated at 70°C for 15 min, and then treated with RNaseH for 20 min at 37°C. The resulting cDNAs were then immediately used for PCR amplification.

cDNAs were first serially diluted and dispensed into the wells of a 96-well plate prior to *env* amplification. At dilutions for which 30% or fewer of the wells are PCR positive, these wells are considered to contain amplicons derived from a single *env* sequence. First-round PCR was performed in a final volume of 20 µL per well, containing 1× High-Fidelity PCR Buffer, 2 mM MgSO₄, 0.2 mM each dNTP, 0.2 µM forward primer Env5out (5′-TAGAGCCCTGGAAGCATCCAGGAAG-3′), 0.2 µM reverse primer Env3out (5′-TTGCTACTTGTGATTGCTCCATGT-3′), 0.05 units/µL Platinum Taq DNA Polymerase, High Fidelity (Invitrogen), and 2 µL of diluted cDNA. Thermocycling conditions were as follows: initial denaturation at 94°C for 2 min; 35 cycles of denaturation at 94°C for 15 s, annealing at 55°C for 30 s, and extension at 68°C for 4 min, followed by a final extension at 68°C for 10 min. Two microliters of the first-round PCR products was then subjected to a second-round PCR using primers targeting the gp140 region: M1bis (5′-CCACCACTCTATTTTGTGCATCA-3′) and gp41-revMam (5′-GGTGGTAGCTGAAGAGGCACA-3′). This second PCR was carried out under the same conditions as the first one, except that it included 45 amplification cycles. Amplicon size (approximately 2 kb) and concentration were then assessed by capillary electrophoresis (Fragment Analyzer, Agilent).

To minimize the risk of cross-contamination, RNA extraction, PCR, and post-PCR steps were conducted in separate, dedicated rooms under PCR clean-room conditions. Reagents were aliquoted in advance, and all steps were performed using dedicated equipment and PCR hoods.

### *Env* sequence analysis

*Env* amplicons were sequenced with the Sanger method (Eurofins TubeSeq Supreme Service) using different primer sets (Table S3). Ambiguous sequences, that is, showing mixed bases at a given nucleotide position or unclear chromatogram peaks, were excluded from further analysis. Sequence alignments and reconstruction were carried out using CodonCode Aligner, while sequence analyses were performed with BioEdit and Unipro UGENE.

Whether *envs* in each plasma sample derive from one or multiple transmission events was analyzed as previously described (20, 100). Briefly, the diversity and differences in nucleotide composition of *env* sequences in each plasma sample were first visualized using *Highlighter* plots (www.hiv.lanl.gov). *Highlighter* was also used to detect APOBEC-mediated mutations. The Poisson-Fitter v2 tool (www.hiv.lanl.gov) was then used to calculate the time since the transmission event and distribution frequencies of HDs. The tool also allows evaluating the extent to which this distribution conforms to a Poisson mutation distribution model (goodness-of-fit between theoretical and observed HD values), and whether *env* sequences have evolved in a star-like phylogeny. Further analyses of the phylogenetic relationships among the *env* sequences were conducted using the Genotoul bioinformatics platform (https://bioinfo.genotoul.fr/). Sequences were aligned with MAFFT, and phylogenetic trees were constructed by the maximum-likelihood method with IQ-TREE, incorporating the best-fit evolutionary model as estimated by the software (F81 + F model), and rooted on a panel of HIV *env* sequences of the same subtype, or at the midpoint. Tree visualization was performed using iTOL (https://itol.embl.de/). Genetic distance was calculated with IQ-TREE and expressed in number of nucleotide mutations for a sequence of 2,000 bp:


$$\text{genetic distance}=\frac{\text{number of nucleotide mutations}}{\text{sequence length (≈ 2,000 bp)}}$$

The presence of recombination within samples was analyzed using GARD (www.datamonkey.org/GARD/), RIP, and RAPR (www.hiv.lanl.gov) tools. Parameters describing the complexity of *env* populations, such as normalized Shannon entropy (H_SN_), nucleotide diversity (π_e_), and Hill numbers of order *q,* were calculated based on the formulas presented in Table S4 and the previous literature from us (65, 120) and others (101). The different regions of the Envs were delineated based on alignment with the HxB2_K03455 reference sequence. Prediction of potential N-linked glycosylation sites in Envs was done using the NetNglyc server (121), and their localization relative to the different Env regions was determined by comparison with the HxB2 sequence using the N-GlycoSite tool (www.hiv.lanl.gov) (122). The net charge of the Env protein was computed using ProtPi (www.protpi.ch). Relative contents of non-synonymous and synonymous mutations (dN − dS differences) in *env* sequences were calculated using MEGA 11 (www.megasoftware.net).

### Cell culture

HEK 293T cells and TZM-bl cells were described previously (104). PBMCs were isolated from the blood of healthy donors by density gradient centrifugation using Human Pancoll (Pan Biotech). CD4+ T cells and monocytes were then purified from PBMCs using CD4- or CD14-positive selection kits (Miltenyi) according to the manufacturer’s instructions. Monocytes were differentiated into macrophages over 9 days in complete RPMI 1640 medium (i.e*.*, supplemented with 10% fetal bovine serum [FBS], 100 µg/mL streptomycin, 100 U/mL penicillin) containing 25 ng/mL macrophage colony-stimulating factor (M-CSF, Miltenyi). For infection experiments, CD4+ T cells in complete RPMI 1640 medium were activated for 2 days in the presence of phytohemagglutinin (PHA, Thermo Fisher Scientific) (1 µg/mL) and interleukin-2 (IL-2, 300 IU/mL, PeproTech), followed by 3 days in the presence of IL-2 alone. For infection experiments of PBMCs, cells suspended in complete RPMI 1640 medium were stimulated with anti-CD3/CD28 antibodies (2.5 µL/mL) for 48 h (T Cell TransAct from Miltenyi Biotec) in the presence of 20 IU/mL IL-2.

Tonsil mononuclear cells were isolated from children’s palatine tonsils, which were used shortly after surgery and handled on ice in phosphate-buffered saline (PBS) containing 5% FBS, 2 mM L-glutamine, 100 µg/mL streptomycin, 100 U/mL penicillin, and 0.1 mg/mL gentamicin. They were cut into small fragments of approximately 3 mm³ and gently pressed through a 40 µm filter to obtain a cell suspension and remove residual tissue. Mononuclear cells were isolated from the suspension by density gradient centrifugation using Human Pancoll (Pan Biotech). TMCs were then immediately inoculated with viruses after being resuspended in RPMI 1640 medium supplemented with 15% FBS, 1 mM sodium pyruvate, 0.1 mM non-essential amino acids, 2 mM L-glutamine, 0.1 mg/mL gentamicin, and 0.1 mg/mL ampicillin.

### Virus production

The recombinant virus populations expressing Envs from plasma samples were prepared as previously described (104, 123). Briefly, following reverse transcription and first amplification PCR using primers, gp160-5out (5′-GGCTTAGGCATCTCCTATGGCAGGAAGAAG-3′) and gp160-3out (5′-GGTCTTAAAGGTACCTGAGGTCTGACTGGA-3′), gp140 *env* sequences were amplified by nested-PCR using the Phusion Expand High Fidelity polymerase (ThermoScientific) and the same primers used for SGA (M1bis and gp41-revMam). Mixes of two or three pooled PCR products were then co-transfected with NheI-linearized pNL43-Δenv-Nef (or Luc2) vector DNA in HEK 293T cells. After 48 h of transfection, viruses in the cell culture supernatants were titrated on TZM-bl cells (Luciferase Assay System kit, Promega) (Nef viruses) or primary CD4+ T cells (Luc2 viruses) and measured for their Gag p24 content (Lenti-X p24 Rapid Titer Kit, Takara). Luc-2-expressing virus populations were used in the infection assays of MDMs (Fig. 6C) or T cells (Fig. S2). The other infections (of TZM-bl cells or T cells in Fig. 6A, B and D; Fig. 7) were done with Nef-expressing viruses. Viruses were stored at −80°C until use.

### Virus infection assays on primary cells

PBMCs and TMCs were plated into round-bottom 96-well plates (1.10^6^ cells/well) in complete RPMI 1640 (as described above) and then inoculated with constant amounts of viruses (approximately 10 ng of p24). Plates were spinoculated at 2,000 × *g* for 2 h at 4°C and then transferred at 37°C. After 16 h of incubation, the medium was changed, and the cells were incubated at 37°C for an additional 4 days. The Gag p24 protein content in the culture supernatant was then measured using the Lenti-X p24 Rapid Titer Kit (Takara). In experiments to determine the coreceptor usage of viruses, activated CD4TL in complete RPMI 1640 medium were infected with constant doses of Luc2-expressing viruses (1 ng of p24) into round-bottom 96-well plates (2.10^5^ cells/well), in the presence or absence of 10 µM MVC, 10 µM AMD3100, or a mixture of both antagonists at 10 µM each. Infectivities were measured 48 h later in the cell lysates by measuring the luciferase activity (Luciferase Assay System kit, Promega). To determine macrophage-tropism, MDMs in flat-bottom 96-well plates (2.10^5^ cells/well) were infected with equal infectious doses of viruses (50,000 RLU measured in CD4TL), incubated at 37°C for 48 h, and infectivities were determined by measuring the luciferase activity (Luciferase Assay System kit, Promega).

### Neutralization assays

The sensitivity of Env-pseudotyped viruses to neutralization by recombinant sCD4 (PeproTech) or bNAbs was studied as follows. Equivalent infectious doses of viruses (1–3 × 10^6^ RLU in TZM-bl cells) in flat-bottom 96-well plates containing complete DMEM (104) were incubated at 37°C for 1 h with 300 nM sCD4 or with serial dilutions of bNAbs (which were produced and purified as described in our previous work [104]). Then, TZM-bl cells were added to the virus-inhibitor mixtures, and incubation was pursued for 48 h. Percent infectivity in the presence of sCD4 or bNAbs, compared to infectivity in the absence of inhibitors (100% infectivity), was calculated as detailed previously (104). In the experiments with bNAbs, results were fitted to a sigmoidal dose–response model with a variable slope. IC₅₀ values were determined from the inhibition curves using GraphPad Prism 10.

### Statistics

All statistical analyses were performed using GraphPad Prism 10. The tests used, selected according to the experimental data presented, are detailed in the figure legends.

## Data Availability

Env nucleotide sequences isolated from the 22 plasma samples have been deposited in GenBank (https://www.ncbi.nlm.nih.gov/genbank/) with the following accession numbers: PX395398–PX395406 (RP1), PX395050–PX395061 (RP2), PX395062–PX395077 (RP4), PX395078–PX395088 (RP5), PX395089–PX395105 (RP6), PX395106–PX395123 (RP7), PX395124–PX395141 (RP8), PX395142–PX395158 (RP9), PX395159–PX395172 (RP10), PX395173–PX395192 (RP11), PX395193–PX395207 (RP17), PX395208–PX395232 (RP18), PX395233–PX395247 (RP19), PX395248–PX395260 (RP20), PX395261–PX395277 (XP1), PX395278–PX395291 (XP2), PX395292–PX395303 (XP4), PX395304–PX395324 (XP6), PX395325–PX395334 (XP7), PX395335–PX395349 (XP8), PX395350–PX395368 (XP9), and PX395369–PX395397 (XP10).

## SUPPLEMENTAL MATERIAL

Supplemental material jvi.00067-26-s0001.pdf Tables S1 to S4; Fig. S1 to S10.

ASM does not own the copyrights to Supplemental Material that may be linked to, or accessed through, an article. The authors have granted ASM a non-exclusive, world-wide license to publish the Supplemental Material files. Please contact the corresponding author directly for reuse.
