# Supplementary material for: Genotypic and phenotypic characteristics of CXCR4-using transmitted/founder HIV-1 envelope glycoproteins
Source: J Virol. 2026 May 14;100(6):e00067-26. doi: 10.1128/jvi.00067-26 (PMC13288616; doi:10.1128/jvi.00067-26)
Supplement: Supplemental material — Tables S1 to S4; Fig. S1 to S10. [file jvi.00067-26-s0001.pdf]

Supplementary Information for:

Genotypic and Phenotypic Characteristics of CXCR4-using Transmitted/Founder HIV-1 Envelope Glycoproteins

Dorine Martres, Théo Montagné, Sandra Pani, Nicolas Jeanne, Marie Armani-Tourret, Mary Requena, Camille Vellas, Stéphanie Raymond, Valérie Lorin, Yohan Gallois, Bénédicte Puissant-Lubrano, Hugo Mouquet, Pierre Delobel, Jacques Izopet, Bernard Lagane

	Sample	Fiebig stage	HIV-1 Subtype	Sex	Transmission mode*	Tropism CD4TL	Tropism U87	RLU on U87 CCR5+	RLU on U87 CXCR4+	Ratio RLU U87 CCR5/ CXCR4
	RP1	II-III	B	Male	MSM	R5	R5	5.39	1.89	3162
	RP2	II-III	B	Male	Bisexual	R5	R5	7.36	1	2290868
	RP3	II	B	Male	MSM	R5	R5	5.72	1.14	38019
	RP4	II	B	Male	Bisexual	R5	R5	7.18	1.98	158489
**$R5_{I-III}$**	RP5	II	B	Male	MSM	R5	R5	7.65	3.55	12589
	RP17	I	CRF02	Male	MSM	R5	R5	5.85	1.6	17783
	RP18	II-III	B	Male	MSM	R5	R5	6.37	1.7	46774
	RP19	II-III	CRF02	Male	MSM	R5	R5	6	1.6	25119
	RP20	II-III	B	Male	MSM	R5	R5	4.81	1.7	1288
	RP6	V	B	Male	MSM	R5	R5	6.83	1.72	128825
	RP7	V	B	Male	MSM	R5	R5	6.83	4.12	513
	RP8	IV-V	B	Male	MSM	R5	R5	7.66	2.61	112202
**$R5_{IV-V}$**	RP9	IV-V	B	Male	MSM	R5	R5	6.18	1.14	109648
	RP10	IV	B	Male	MSM	R5	R5	6.04	1.24	63096
	RP11	IV-V	CRF02	Male	MSM	R5	R5	6.64	1.6	109648
	RP13	IV-V	B	Male	MSM	R5	R5	6.09	1.7	24547
	XP1	II-III	B	Female	Heterosexual	R5X4	R5X4	6.84	7.13	0.51
	XP2	V	B	Male	IDU	R5X4	R5X4	6.58	6.85	0.54
	XP4	IV-V	B	Male	MSM	R5X4	R5X4	7.06	6.60	2.88
	XP6	IV-V	B	Male	MSM	$_{R5}X4$	R5X4	5.94	7.05	0.08
**XP**	XP7	IV-V	CRF02	Male	MSM	X4	X4	1.9	6.48	0.00
	XP8	V	B	Female	Heterosexual	$R5_{X4}$	R5X4	6.49	5.46	10.72
	XP9	VI	B	Male	MSM	$_{R5}X4$	R5X4	3.06	5.7	0.00
	XP10	IV-V	B	Male	Bisexual	$_{R5}X4$	R5X4	5.78	7.08	0.05

*MSM: Men having sex with men, IDU: injecting drug users
Genotype for CCR5 mutation Δ32 was done on samples XP1, XP2 and XP4: XP1 and XP4 were homozygous wild type, and XP2 was heterozygous.

Table S1. Characteristics and tropism of primary HIV-1 infection samples. The classification of XP virus tropism in T cells was based on their sensitivity to inhibition by MVC, AMD3100 or both, as shown in Fig. S2. X4 indicates that the virus is inhibited by AMD alone but not by MVC. $R5_{X4}$ indicates that the virus is more efficiently inhibited by MVC than by AMD. The samples (XP1-4) that were inhibited by AMD alone but only partially (< 90 % inhibition) and for which further inhibition required the combination of AMD with MVC were identified as R5X4. The remaining samples that were more efficiently inhibited by AMD (>> 90 %) were classified as $_{R5}X4$.

V1

	Relative position	1	2	3	4	5	6	7	8	9	10	11	12	13	14	15	16	17	18	19	20	21	22	23	24	25	26	27	28	29	30	31	32	33	34	35	36	37	38	39	40	41	42	43	44	45	46	47	48	49
	HxB2	C	T	D	L	-	-	-	-	-	-	-	-	-	K	N	D	-	-	-	-	T	N	T	N	S	-	-	-	-	-	S	S	G	R	M	I	M	E	K	G	-	-	E	I	K	N	-	-	-
**R5 I-III**	RP1	C	T	E	P	S	-	-	-	-	-	-	-	N	K	T	S	-	-	-	-	T	D	N	T	S	-	-	-	-	-	N	A	N	N	T	S	R	D	W	K	E	I	G	M	T	N	-	-	-
	RP2	C	T	N	L	T	-	-	-	-	-	-	K	N	A	T	S	-	-	-	-	T	N	S	T	N	-	-	-	-	-	D	T	G	H	N	N	I	T	L	G	E	G	G	M	K	N	-	-	-
	RP4	C	T	D	K	V	G	N	I	-	-	-	T	N	N	N	S	S	F	-	-	T	N	S	S	W	-	-	-	-	-	D	K	-	-	-	-	M	E	T	G	-	-	E	I	K	N	-	-	-
	RP5	C	T	E	P	L	R	K	N	D	T	-	N	S	T	N	N	T	S	I	-	A	N	N	T	S	-	-	-	-	-	S	G	-	-	-	-	W	E	E	I	-	-	E	M	K	N	-	-	-
	RP17-1	C	T	N	P	N	R	R	N	T	T	L	N	N	T	N	S	T	-	-	-	F	N	S	T	N	-	-	-	-	-	N	A	-	-	-	-	Y	E	-	-	-	-	D	M	K	N	-	-	-
	RP17-2	C	T	N	P	N	R	K	N	T	T	L	N	N	T	N	S	T	-	-	-	F	N	S	T	N	-	-	-	-	-	N	A	-	-	-	-	Y	E	-	-	-	-	D	M	K	N	-	-	-
	RP18	C	T	N	Y	-	-	-	-	-	-	-	-	-	-	-	-	-	-	S	T	Y	N	N	S	R	-	-	-	-	-	-	-	-	-	-	-	V	G	D	L	R	G	E	V	K	N	-	-	-
	RP19	C	H	S	V	-	-	-	-	-	-	-	-	-	-	N	I	-	-	-	-	T	N	T	T	T	Q	-	-	-	-	N	I	-	-	-	-	T	E	D	I	R	R	E	I	S	N	-	-	-
	RP20	C	T	D	V	-	-	-	-	-	-	-	T	N	S	N	S	T	H	S	N	S	N	S	T	H	-	-	-	N	N	N	A	N	T	T	V	S	D	Q	I	-	-	G	V	K	N	-	-	-
**R5 IV-V**	RP6	C	T	V	A	T	-	-	-	-	-	-	-	N	S	S	S	-	-	-	-	I	N	T	T	V	-	-	-	-	-	S	-	-	-	S	K	I	E	-	-	-	-	G	V	K	N	-	-	-
	RP7	C	T	V	A	T	-	-	-	-	-	-	-	N	S	S	S	-	-	-	-	A	N	S	T	N	-	-	-	-	-	D	-	-	-	N	K	I	E	-	-	-	-	G	V	K	N	-	-	-
	RP8	C	T	E	P	-	-	-	-	-	-	-	-	-	S	N	S	-	-	-	-	T	N	S	T	G	-	-	-	-	-	S	A	N	N	N	S	S	V	W	E	E	I	G	M	T	N	-	-	-
	RP9	C	T	N	I	N	E	K	I	T	T	-	-	G	I	N	T	S	D	T	T	I	N	E	T	N	I	A	K	L	N	N	I	-	-	-	-	T	E	-	-	-	-	E	M	K	N	-	-	-
	RP10-1	C	T	N	V	N	-	-	-	-	-	V	T	N	T	N	V	T	Y	A	P	A	N	S	T	G	-	-	-	-	-	N	M	-	-	-	-	T	E	-	-	-	-	E	I	K	G	-	-	-
	RP10-2	C	I	N	V	-	-	-	-	-	-	-	T	N	T	N	V	-	-	-	-	T	N	S	T	N	-	-	-	-	-	A	T	V	S	P	G	S	E	-	-	-	-	E	V	R	N	-	-	-
	RP11	C	S	D	A	T	N	N	I	T	T	M	P	G	V	N	L	-	-	-	-	-	-	-	-	-	-	-	-	-	-	N	M	-	-	-	-	T	G	-	-	-	-	E	I	K	N	-	-	-
**XP**	XP1	C	T	D	V	-	-	-	-	-	-	-	-	-	V	S	N	-	-	-	-	T	T	N	T	T	-	-	-	-	-	S	N	G	E	G	M	M	E	K	G	-	-	E	I	K	N	-	-	-
	XP2	C	T	D	L	-	-	-	-	-	-	-	K	H	T	N	K	-	-	-	-	T	N	L	T	N	-	-	-	-	-	A	E	-	-	-	E	P	D	-	-	-	-	Q	M	K	N	-	-	-
	XP4	C	T	N	L	-	-	-	-	-	-	-	-	-	-	-	-	-	-	-	-	A	N	S	T	D	-	-	-	-	-	N	M	-	-	-	-	K	E	-	-	-	-	E	M	K	N	-	-	-
	XP6	C	T	D	V	N	-	-	-	-	-	-	T	T	S	S	N	-	-	-	-	T	N	T	S	S	-	-	-	-	-	S	N	S	I	M	K	M	P	L	G	-	-	E	I	K	N	-	-	-
	XP7	C	T	T	V	T	-	-	-	-	-	-	-	-	N	S	T	-	-	-	-	G	N	S	T	D	-	-	-	-	-	S	R	N	I	T	I	G	K	D	M	E	G	E	V	K	N	-	-	-
	XP8	C	T	D	Y	D	-	-	-	-	-	-	-	-	F	N	V	-	-	-	-	T	T	N	A	T	-	-	-	-	-	A	A	N	D	T	G	M	R	G	M	R	G	E	V	K	N	-	-	-
	XP9-R5	C	V	N	V	-	-	-	-	-	-	-	-	-	S	T	S	-	-	-	-	T	N	N	S	S	-	-	-	-	-	N	S	N	-	-	-	Q	E	M	Y	R	N	E	I	K	N	-	-	-
	XP9-X4	C	S	D	Y	-	-	-	-	-	-	-	S	G	N	N	T	-	-	-	-	V	N	S	T	S	-	-	T	N	N	N	R	-	S	L	L	Q	E	N	L	G	-	E	I	K	N	-	-	-
	XP10-1	C	S	D	Y	-	-	-	-	-	-	-	T	G	N	N	A	-	-	-	-	T	N	N	N	N	-	-	G	L	S	N	S	-	S	L	L	E	E	S	K	G	-	E	I	K	N	-	-	-
	XP10-2	C	S	D	Y	-	-	-	-	-	-	-	T	G	N	N	A	-	-	-	-	T	N	N	N	N	-	-	G	L	S	N	S	-	S	L	L	E	E	S	K	G	-	E	I	K	N	-	-	-

V2

Group	Relative position	1 2 3 4 5 6 7 8 9 10	11 12 13 14 15 16 17 18 19 20	21 22 23 24 25 26 27 28 29 30	31 32 33 34 35 36 37 38 39 40	41 42 43 44 45 46 47 48 49 50	51 52 53 54 55 56 57 58 59
	HxB2	C S F N I S T S I R	G K V Q K E Y A F F	Y K L D I I P I - -	- - - - - - D N D T	- - - - - - - - - -	T S Y K L T S C
	RP1	C S F N V T A G I K	D K V Q K E Y A L L	Y K T D I V S I - -	- - - - - - K D G S	- - - - - - - - - -	T E Y M M I H C
	RP2	C S F N I A T G I G	N K R K K E Y A L F	Y N L D L V Q I D G	P - - - - - - - - -	- - - - - - - - - -	G N Y T L I T C
	RP4	C S F N I T T N M R	D E I K K E R A L F	Y K L D I V P I G -	- - - - - - - N S N	- - - - - - - - - -	T S Y R L I S C
	RP5	C S F N V T A G I K	D K V Q K E Y A L L	Y K T D I V S I - -	- - - - - - N N S S	- - - - - - - - - -	N E Y M M I H C
R5 I-III	RP17-1	C S F N I T T E I R	D K T R R E Y A L F	Y K L D I V Q I N D	N E E S N A G N E T	S S A S N G T S N A	S Q Y R L I N C
	RP17-2	C S F N I T T E I R	D K T R R E Y A L F	Y K L D I V Q I N D	N E E S N A G N G T	S S A S N G T S N A	S Q Y R L I N C
	RP18	C S F N V S T S R R	D K M Q K G Y A I F	D K T D V V S I D -	- - - - - G S N S -	- - - - - - - - - -	- S Y M L I N C
	RP19	C S F N T T T V I R	D K K Q S A Y A L F	Y R T D L V S I D -	- - - - S G K N N N	S N - - N N S K G E	G S Y I L T N C
	RP20	C S F N V T T N I R	D Q I Q K A Y A I F	Y N L D I V Q I D -	- - - - - - D N D N	- - - - - - - - - -	S S Y R L I H C
	RP6	C S F N V T T N I R	D K M Q K A Y A L F	Y D L D I V P I - -	- - - - - - D N D N	- - - - - - - - - -	S S Y R L I H C
	RP7	C S F N V T T N I R	D K M Q K A Y A L F	Y D L D I V P I - -	- - - - - - D N D N	- - - - - - - - - -	S S Y R L I H C
	RP8	C S F N V T A G I K	D K V Q K E Y A L L	Y K T D I V S I - -	- - - - - - K N G S	- - - - - - - - - -	S E Y M M I H C
R5 IV-V	RP9	C S F N V T T S I R	D K V K K E Y A L F	Y K L D V M S I - -	- - - - - - A N D S	- - - - - - - - - -	T S Y T L I N C
	RP10-1	C S F N V T T N I R	D K V K T E Y A I F	Y N L D I V Q I E -	- - - - - - D K N N	- - - - - - - - - -	T S Y R I R N C
	RP10-2	C S F N V T T N I R	D K V K K E H A I F	Y S L D I V P I K -	- - - - - - E N N D	S - - - - - - - - -	T S Y R I R S C
	RP11	C S F N T T T E L R	D K T K K M H A L F	Y R L D V V Q I E -	- - - - - - G N T S	- - - - - - - - - -	N M Y T L I N C
	XP1	C S F N I T T N I R	D R V Q K E Y A L F	Y N L D V V P I E -	- - - - - - N K S N	- - - - N S S R N N	T S F R L I S C
	XP2	C S F N V T T S I G	N K M Q K E Y A L F	Y K L D V V P I - -	- - - - - - G D T S	- - - - - - - Q N H	T N Y T L I H C
	XP4	C S F N V T T N I R	D K V Q K E Y A L L	Y K L D I V P I E -	- - - - - - N E N N	S A N G N N S V N S	T N Y R L I S C
	XP6	C S F N I T T D L Q	D K R Q K Q Y A L F	Y A L D V V Q I D -	- - - - - - - - - -	- - - - - - - N D T	- - Y R L T S C
XP	XP7	C S F N V T T E L R	D K K K Q E Y A L F	Y R Q D V V Q I D -	- - - - - - - N S T	S S - - - - - Q N Y	S Q Y M L I N C
	XP8	C S F N T T T S I R	D K I S K E T A L F	Y K L D V V P I - -	- - - - - - D N D N	- - - - - - - - - -	H S Y R L I N C
	XP9-R5	C S F N I T S S I R	D R V H Q N Y A L F	D K F D I V P I T D	K E - - - - E N D T	- - - - - - - R N -	N T Y R L I H C
	XP9-X4	C S F N V T S D I R	D K E R R E Y A L F	Y K L D V V - - - -	- - K L G D D N T N	- - - - - - - - - -	T S Y R L I S C
	XP10-1	C S F N I T T N I Q	D K R Q K G N A L F	Y K L D L V Q M G -	- - - - - - - - - -	- - - - - - - N N G	S Y Y R L I S C
	XP10-2	C S F N I T T N I Q	D K R Q K G N A L F	Y K L D L V Q M G -	- - - - - - - - - -	- - - - - - - N N G	S Y Y R L I S C

V3

	Relative position	1	2	3	4	5	6	7	8	9	10	11	12	13	14	15	16	17	18	19	20	21	22	23	24	25	26	27	28	29	30	31	32	33	34	35	36	37	38	39
	HxB2	C	T	R	P	N	N	N	T	R	K	R	I	R	I	Q	R	G	P	-	-	G	R	A	F	V	T	I	G	K	I	-	G	N	M	R	Q	A	H	C
	RP1	C	T	R	P	N	N	N	T	R	K	S	I	H	I	-	-	V	P	-	-	G	G	A	F	Y	A	T	G	D	I	I	G	D	I	R	Q	A	H	C
	RP2	C	T	R	P	N	N	N	T	R	K	S	I	N	L	-	-	G	P	-	-	G	R	T	I	Y	A	T	G	D	I	I	G	D	I	R	Q	A	H	C
	RP4	C	T	R	P	N	N	N	T	R	R	S	I	R	I	-	-	G	P	-	-	G	-	S	Y	F	A	T	G	D	I	I	G	D	I	R	K	A	Y	C
	RP5	C	T	R	P	N	N	N	T	R	K	S	I	H	I	-	-	A	P	-	-	G	R	A	F	Y	A	T	G	E	I	I	G	D	I	R	Q	A	H	C
**R5 II-III**	RP17-1	C	T	R	P	N	N	N	T	R	K	G	V	H	I	-	-	A	P	-	-	G	H	A	F	Y	I	N	T	E	I	V	G	D	I	R	K	A	Y	C
	RP17-2	C	T	R	P	N	N	N	T	R	K	G	V	H	I	-	-	A	P	-	-	G	H	A	F	Y	I	N	T	E	I	V	G	D	I	R	K	A	Y	C
	RP18	C	T	R	P	N	N	N	T	R	Q	G	I	H	I	-	-	G	P	-	-	G	R	A	F	Y	A	R	T	E	I	T	G	D	I	R	Q	A	H	C
	RP19	C	T	R	P	S	N	N	T	R	K	S	I	R	I	-	-	G	P	-	-	G	Q	T	F	Y	A	Y	G	D	M	I	G	D	I	R	Q	A	F	C
	RP20	C	A	R	P	S	N	N	T	R	K	S	I	P	I	-	-	G	P	-	-	G	R	A	F	Y	A	T	G	E	I	T	G	D	I	R	Q	A	H	C
	RP6	C	T	R	P	S	N	N	T	R	K	S	I	H	I	-	-	G	P	-	-	G	R	A	F	Y	A	T	G	E	I	I	G	N	I	R	Q	A	H	C
	RP7	C	T	R	P	S	N	N	T	R	K	S	I	P	I	-	-	G	P	-	-	G	R	A	F	Y	A	T	G	D	I	I	G	D	I	R	Q	A	H	C
	RP8	C	T	R	P	N	N	N	T	R	K	S	I	H	I	-	-	A	P	-	-	G	G	A	F	Y	A	T	G	D	I	I	G	D	I	R	Q	A	H	C
**R5 IV-V**	RP9	C	T	R	P	S	N	N	T	R	R	G	I	H	M	-	-	A	V	-	-	G	R	A	F	Y	A	T	G	Q	I	I	G	D	I	R	Q	A	H	C
	RP10-1	C	I	R	P	N	N	N	T	R	K	S	I	H	I	-	-	A	P	-	-	G	R	A	F	Y	A	T	G	N	I	I	G	D	I	R	K	A	Y	C
	RP10-2	C	T	R	P	N	N	N	T	R	K	S	I	H	I	-	-	A	P	-	-	G	R	A	F	Y	A	T	G	D	I	I	G	D	I	R	K	A	H	C
	RP11	C	V	R	P	N	N	N	T	R	K	S	I	T	V	-	-	G	P	-	-	G	K	V	F	W	-	T	N	D	I	I	G	N	I	R	Q	A	H	C
	XP1	C	T	R	P	N	N	N	T	S	K	G	I	H	T	-	-	G	P	-	-	G	R	A	F	I	A	R	R	T	I	I	G	N	I	R	Q	S	H	C
	XP2	C	T	R	P	N	N	N	T	R	K	S	I	H	M	-	-	G	A	-	-	G	R	A	F	Y	T	N	G	E	I	I	G	N	I	R	R	A	Y	C
	XP4	C	T	R	P	G	N	N	T	R	K	M	I	H	I	-	-	G	P	-	-	G	R	A	W	Y	A	T	D	K	I	I	G	N	I	R	K	A	H	C
	XP6	C	E	R	P	N	N	N	T	R	K	R	I	S	I	-	-	G	P	-	-	G	R	A	F	R	T	T	G	Q	I	I	G	D	I	R	Q	A	H	C
**XP**	XP7	C	T	R	P	G	N	N	T	R	R	R	V	R	I	-	-	G	I	G	P	G	Q	A	F	H	A	T	G	D	I	I	G	D	I	R	Q	A	Y	C
	XP8	C	T	R	P	G	N	N	T	S	K	G	I	H	I	-	-	G	P	-	-	G	R	A	F	Y	A	T	E	R	I	I	G	D	I	R	R	A	H	C
	XP9-R5	C	T	R	P	N	N	N	T	R	R	S	I	N	I	-	-	G	P	-	-	G	R	A	F	Y	G	T	-	D	I	I	G	D	I	R	Q	A	H	C
	XP9-X4	C	T	R	P	N	N	N	T	S	K	R	I	S	I	-	-	G	P	G	R	G	R	A	I	Y	A	T	E	R	I	I	G	D	I	R	K	A	H	C
	XP10-1	C	T	R	P	N	N	N	T	R	K	G	I	H	I	-	-	G	P	-	-	G	R	R	W	Y	T	T	D	R	I	V	G	D	I	R	K	A	H	C
	XP10-2	C	T	R	P	N	N	N	T	R	K	G	I	H	I	-	-	G	P	-	-	G	R	R	W	Y	T	T	D	R	I	V	G	D	I	R	K	A	H	C

V4

Group	Relative position	1 2 3 4 5 6 7 8 9 10	11 12 13 14 15 16 17 18 19 20	21 22 23 24 25 26 27 28 29 30	31 32 33 34 35 36 37 38 39 40	41 42 43 44 45 46 47 48 49 50	51 52
	HxB2	C **N** S T Q L F **N** S T	W F **N** S T W S - - -	- - - - T E - - - -	- - G S **N** - - - - N	T E G S D T - I T L	P C
R5 I-III	RP1	C **N** T S K L - - - -	- F **N** S S W I H N N	G **N** W T N **N** - - - -	- - G T M V W D T N	D **N** - - D T - I I L	P C
	RP2	C **N** T T E L - - - -	- F **N** S T W S - - -	- - - - T **N** - - - -	- - S T R - - - - **N**	- E T S E K - I T L	P C
	RP4	C N T T Q L - - - -	- F **N** S T W P - - -	- - - - L **N** - - - -	- - S T G - **N** I S N	L **N** E T - - - I I L	P C
	RP5	C D T T P L - - - -	- F **N** S S W T - - -	- - - - K N G I **N** W	T L K G N - T N D R	T T S **N** D T - I I L	Q C
	RP17-1	C **N** T S G L - - - -	- F **N** S T W T - - -	- - - - W **N** - - - -	- - D T A - S S **N** H	T E S **N** D T - I T L	Q C
	RP17-2	C **N** T S G L - - - -	- F **N** S T W T - - -	- - - - W **N** - - - -	- - D T A - S S **N** H	T E S **N** D T - I T L	Q C
	RP18	C D T T K L - - - -	- F **N** S T W - - - -	- - - - G **N** - - - -	- - D T E S K S F Q	G **N** - - D T - I T L	P C
	RP19	C **N** T T A L - - - -	- F **N** S T W Y I **N** G	S - - - S D - - - -	- - **N** S S - S D **N** S	S **N** A T S F - I I L	P C
	RP20	C **N** T T Q L - - - -	- F **N** S T G Y - - -	- - - - G T - - - -	- - K E V - - - - **N**	S T S **N** S T - I T L	Q C
R5 IV-V	RP6	C **N** S T Q L - - - -	- F **N** S T W L - - -	- - - - F **N** - - - -	- - S T G - **N** D T T	E **N** G T - - - I T L	Q C
	RP7	C **N** T T Q L - - - -	- F **N** S T W **N** - - -	- - - - G T - - - -	- - **N** G T R Q A D **N**	T T G **N** N T - I T L	Q C
	RP8	C **N** T S K L - - - -	- F **N** S S W T K **N** S	S N - - S **N** - - - -	- - W T M K **N** N T N	D **N** T T D P - I T L	P C
	RP9	C **N** T S Q L - - - -	- F **N** N T W Y - - -	- - - - K **N** - - - -	- - K T W - Q Y T N	T E D P - - - I I L	P C
	RP10-1	C **N** T S K L - - - -	- F **N** S T W - - S K	S T G - Y **N** - - - -	- - G T - - - - - K	E **N** - - D T - I T L	P C
	RP10-2	C **N** T S K L - - - -	- F **N** S T W - - N **N**	S T W - H **N** - - - -	- - G T W T N G N G	E **N** - - D T - I T L	P C
	RP11	C **N** T S E L - - - -	- F **N** S S W - - - -	- - - - A **N** - - - -	- - G T E V T **N** G T	E N R N E **N** - I T L	P C
XP	XP1	C **N** S T Q L - - - -	- F **N** S T W **N** - - -	- - - - M **N** - - - -	- - S T E - G S S N	T E G N D I - I T L	P C
	XP2	C D T S Q L - - - -	- F **N** S T W N V T Q	- - - - P **N** - - - -	- - A T Q I K G S T	G **N** N T **N** G T I I L	H C
	XP4	C **N** S T Q L - - - -	- F **N** S T W S - - -	- - - - K S - - - -	- - N G **N** W T S **N** A	T E G S D T - I T L	Q C
	XP6	C **N** T T Q L - - - -	- F **N** S T W F - - -	- - - - K **N** - - - -	- - D T D N N **N** S S	E D S T D T - I T L	P C
	XP7	C **N** T T A L - - - -	- F **N** S T W S - - -	- - - - E K - - - -	- - S **N** A - T N G L	E S N **N** D T I I T L	Q C
	XP8	C **N** T T G L - - - -	- F **N** S T W I - **N** G	T - - - Q **N** - - - -	- - N T G - N V **N** D	T N G A - - - I I L	P C
	XP9-R5	C **N** T T P L - - - -	- F **N** S T W **N** M T D	N - - - W **N** - - - -	- - S T G - T **N** D T	R D D T G V - I T L	Q C
	XP9-X4	C **N** T T Q L - - - -	- F **N** S T W T - - -	- - - - S N - - - -	- - **N** I - - - - T **N**	T T E G E I - I T L	P C
	XP10-1	C **N** T T K L - - - -	- F **N** S S W T G N G	K T W - - **N** - - - -	- - R T - - - D E L	**N** N T S E I - I T L	P C
	XP10-2	C **N** T T K L - - - -	- F **N** S S W T G N G	K T W - - **N** - - - -	- - R T - - - D E L	**N** N T S E I - I T L	P C

V5

Group	Relative position	1	2	3	4	5	6	7	8	9	10	11	12	13	14	15	16	17	18	19	20
	HxB2	-	-	-	-	-	-	S	N	-	-	-	N	E	S	E	I	F	R	P	G
R5 I-III	RP1	V	K	Q	**N**	S	T	E	**N**	S	T	G	**N**	R	T	E	I	F	R	P	G
	RP2	-	-	-	-	**N**	R	T	N	-	-	-	**N**	N	T	E	T	F	R	P	G
	RP4	-	-	-	-	-	-	Q	I	-	-	-	**N**	D	T	E	T	F	R	P	A
	RP5	-	-	-	N	D	N	N	A	**N**	Q	-	S	S	T	E	V	F	R	P	G
	RP17-1	-	-	-	-	-	-	**N**	T	-	-	-	S	G	**N**	E	T	F	R	P	G
	RP17-2	-	-	-	-	-	-	**N**	T	-	-	-	S	K	**N**	E	T	F	R	P	G
	RP18	-	-	-	-	-	-	N	**N**	-	-	-	T	T	K	E	T	F	R	P	G
	RP19	-	-	-	T	E	N	S	N	A	E	-	**N**	S	T	E	T	F	R	P	G
	RP20	-	-	-	H	N	N	G	**N**	N	T	-	**N**	K	T	E	V	F	R	P	G
R5 IV-V	RP6	-	-	-	H	E	**N**	G	T	**N**	T	-	T	N	T	E	V	F	R	P	G
	RP7	-	-	-	H	E	**N**	E	T	**N**	T	-	T	N	T	E	V	F	R	P	G
	RP8	-	V	R	Q	**N**	S	T	E	-	-	-	**N**	N	T	E	V	F	R	P	G
	RP9	-	-	-	-	-	-	-	**N**	-	-	-	G	T	T	E	I	F	R	P	G
	RP10-1	-	-	-	-	-	N	N	Y	-	-	-	**N**	G	T	E	I	F	R	P	G
	RP10-2	-	-	-	-	N	**N**	N	S	-	-	-	N	G	**N**	E	T	F	R	P	G
	RP11	S	G	S	**N**	S	S	**N**	E	-	-	-	T	S	E	E	T	F	R	P	G
XP	XP1	-	-	-	-	N	**N**	D	T	-	-	-	**N**	N	T	E	V	F	R	P	G
	XP2	-	-	-	-	G	**N**	K	T	-	-	-	**N**	E	T	E	I	F	R	P	G
	XP4	-	-	-	-	-	-	**N**	T	-	-	-	S	N	T	E	T	F	R	P	G
	XP6	-	-	H	**N**	E	S	E	S	-	-	-	**N**	R	T	E	V	F	R	P	G
	XP7	-	-	-	-	-	G	N	**N**	-	-	-	S	T	**N**	E	T	F	R	P	A
	XP8	-	-	G	**N**	E	S	G	S	-	-	-	**N**	N	T	E	I	F	R	P	E
	XP9-R5	-	-	-	Y	G	**N**	N	T	-	-	-	**N**	N	T	E	I	F	R	P	G
	XP9-X4	-	-	-	-	-	-	S	**N**	-	-	-	S	T	**N**	E	T	F	R	P	G
	XP10-1	-	-	-	-	K	E	G	T	-	-	-	**N**	N	T	E	V	F	R	P	T
	XP10-2	-	-	-	-	K	E	G	T	-	-	-	**N**	N	T	E	V	F	R	P	T

Table S2. Position of PNGS in gp120's variable loops. Consensus sequences of gp120's V1 (A), V2 (B), V3 (C), V4 (D) and V5 (E) in groups $R5_{I-III}$, $R5_{IV-V}$ and XP were aligned with the HxB2_K03455 sequence and analyzed with the N-GlycoSite tool (www.hiv.lanl.gov) to determine the position of PNGS (N-X-S/T sequon) shown in red.

Primer	Sequence (5'-3')
**350-**	GAGCAGTTTT TTATTTCTCC
**350-RP17**	GAGCAGTTTTTCATGTCCTC
**700-**	CTTTAGAATC GCAAAACCAGC
**700+**	GCTGGTTTTG CGATTCTAAAG
**787-**	TGTGTGCATTGTACTGTGCTGACATT
**1250+**	GCAGAATAAAACAAATTATAAAC
**1291-**	GGGAGGGGCATACATTGC
**1291+**	GCAATGTATGCCCCTCCC
**1450-**	ATAATTCACT TCTCCAATTGTC
**1450+**	GACAATTGGA GAAGTGAATTAT

Table S3. Sanger sequencing primers

**Shannon entropy normalized to log(N)**	H_{SN}	$\mathbf{H_{SN}(p) = -\sum_{i=1}^{H} p_i \log(p_i) / \log(N)}$
**Hill Numbers**	${}^{q}D(p)$	${}^{q}D(p) = \left(\sum_{i=1}^{H} p_i^q\right)^{1/(1-q)}$ ${}^{q}H(p) = \sum_{i=1}^{H} p_i^q$
**Population nucleotide diversity**	π	$\pi = \sum_{i=1}^{H}\sum_{j=1}^{H} p_i d_{ij} p_j$

D = matrix of haplotypes pair-wise genetic distances
${}^{q}D$ = Hill number of order q
d_{ij} = genetic distance between the *i*-th and *j*-th haplotype
H = number of haplotypes
N = total number of clones sampled
p = number of polymorphic sites
p_i = population frequency of the *i*-th haplotype in the viral quasispecies
q = order of Hill numbers

Table S4. Diversity and complexity parameters formulae

Fig. S1

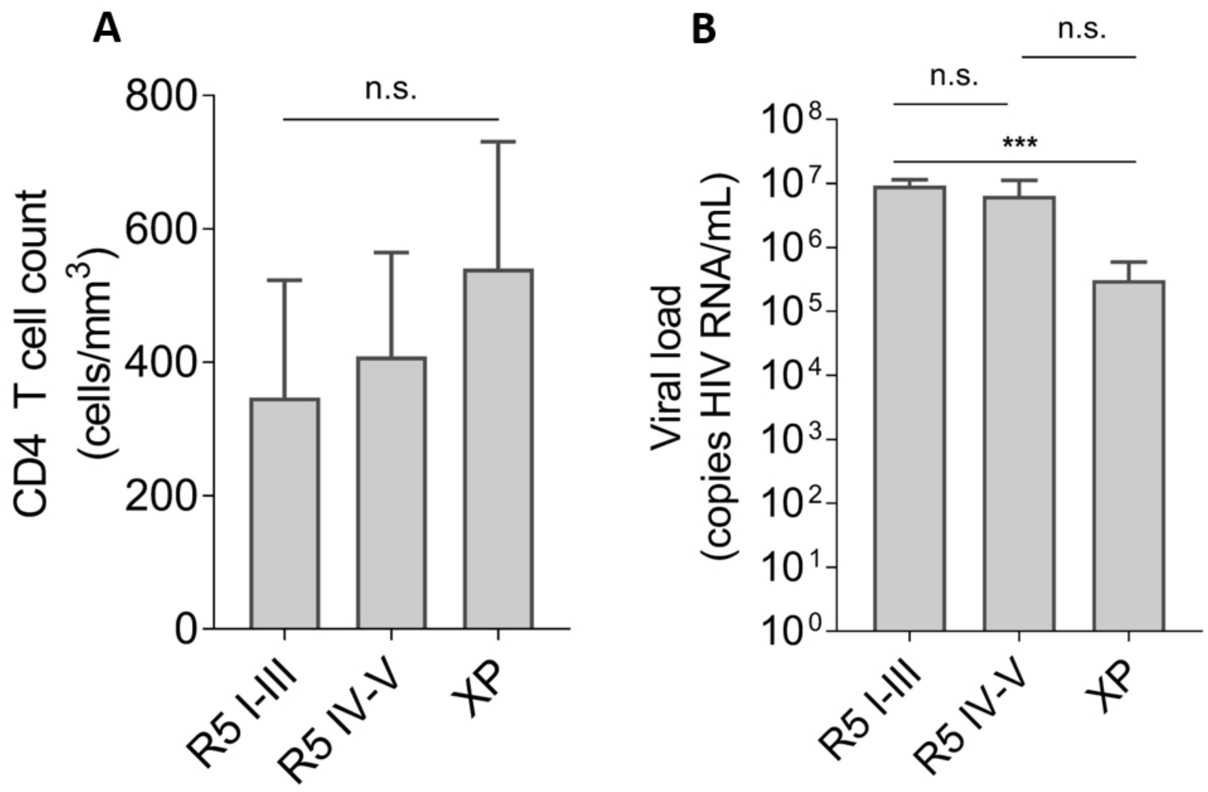

Fig. S1. Blood CD4+ T cell count and viral load in individuals of the study. Blood CD4+ T cell count (A) and viral load (B) in individuals of groups $R5_{I-III}$, $R5_{IV-V}$ or XP, determined at the time of diagnosis and plasma isolation. Statistics: Mann-Whitney *U*-test: ***, $p < 0.001$; n.s., non-significant.

Fig. S2

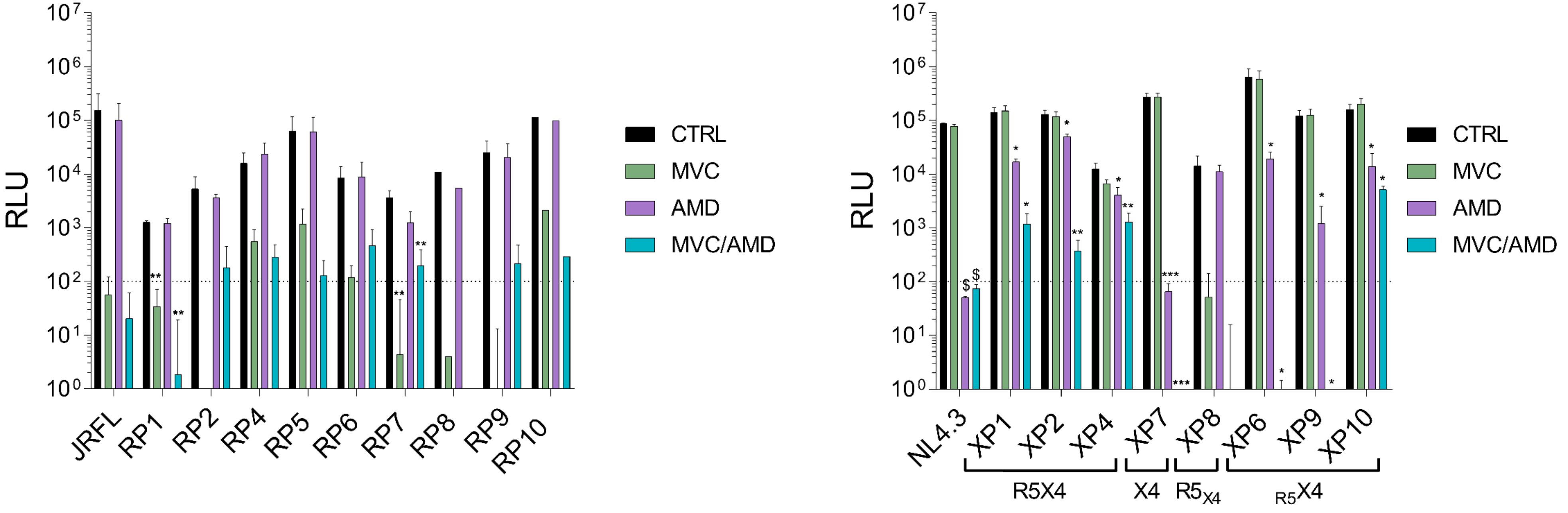

Fig. S2. Sensitivity of Envs from groups RP (I-III and IV-V) and XP to inhibition by coreceptor antagonists. PHA/IL-2-activated CD4TL from healthy donors were inoculated with 1 ng p24 of Luc2-expressing recombinant viruses pseudotyped with the indicated plasma Env populations, in the absence (CTRL) or presence of 10μM Maraviroc (MVC, CCR5 antagonist), AMD3100 (AMD, CXCR4 antagonist) or a mixture of both antagonists at 10μM each. Infectivities expressed as RLU were measured 48 h later in the cell lysates. Statistics (when n ≥ 3): unpaired t-test, comparison with CTRL within each sample. *, **, *** and $ indicate $p < 0.05$, $p < 0.01$, $p < 0.001$ and $p < 0.0001$, respectively. In each panel, the dashed line represents the background signal obtained under uninfected conditions.

Fig. S3

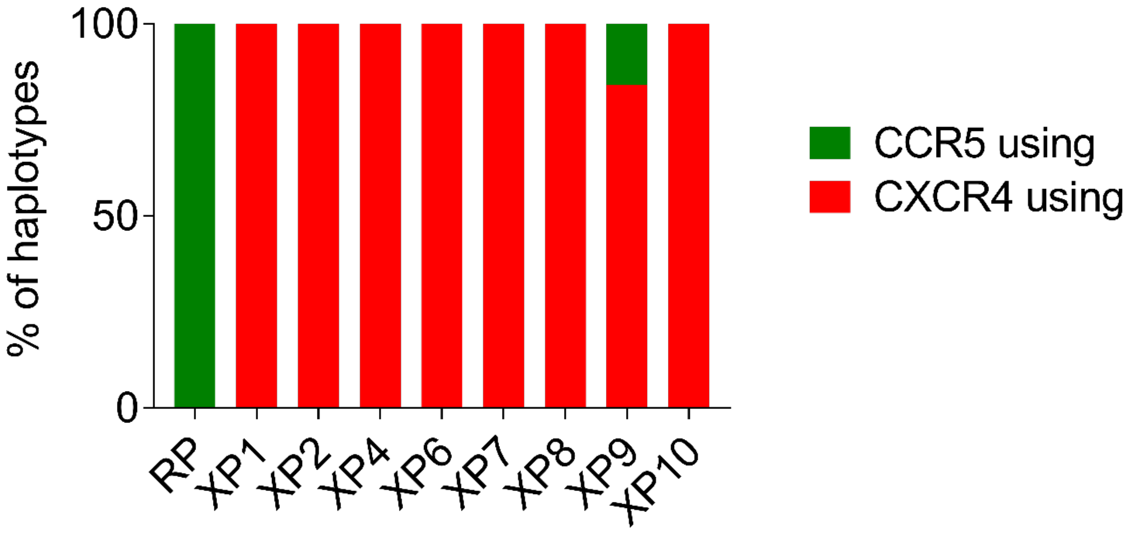

Fig. S3. Genotypic prediction of Env coreceptor usage using Geno2Pheno. Viral tropism (*i.e.*, coreceptor usage) of Envs in plasma samples of RP and XP groups was predicted from their nucleotidic sequences obtained by SGA, with Geno2Pheno[coreceptor] (www.coreceptor.geno2pheno.org), using the original g2p coreceptor prediction method and the recommendations from the European Consensus Group on clinical management of HIV-1 tropism testing (FPR cutoff of 10% FPR). Results represent the percentages of haplotypes predictive of pure R5 tropism or CXCR4 usage (X4 or R5X4 tropism). Except for XP9 and XP10, *env* sequences in the other XP samples had the same FPR values: XP1 1.7%, XP2 3.8%, XP4 1.7%, XP6 1.3%, XP7 0.1%, XP8 1.7 %. In XP9, X4 sequences and R5 sequences had an FPR of 0.1% and 38%, respectively. For XP10, all sequences had an FPR value of 2.9%, except haplotypes 2B12 and 2E10, which had values of 3.4% and 6% (see Fig. 3D). For the R5 samples (including the R5 cluster in XP9), the FPR values were equal to 10 (RP4) or substantially higher (> 20).

Fig. S4

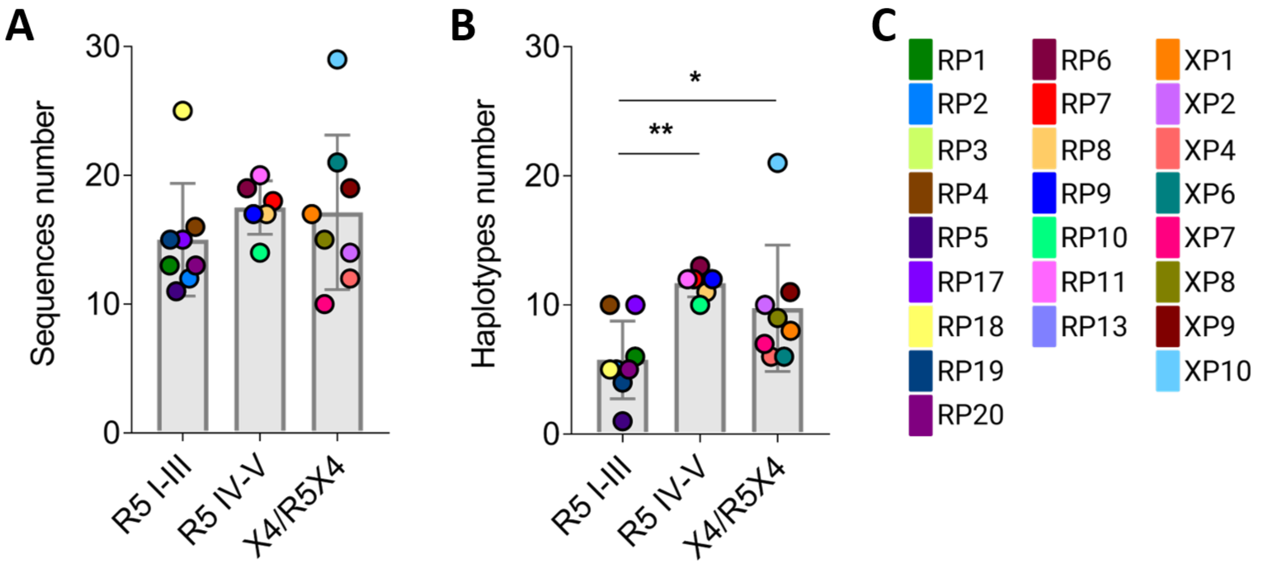

Fig. S4. Number of *env* sequences and haplotypes in plasmas. Number of *env* sequences (A) and corresponding haplotypes (B) obtained by SGA from plasma samples of individuals in groups $R5_{I-III}$, $R5_{IV-V}$ and XP. (C) Color code of plasma samples used throughout the article.

Fig. S5

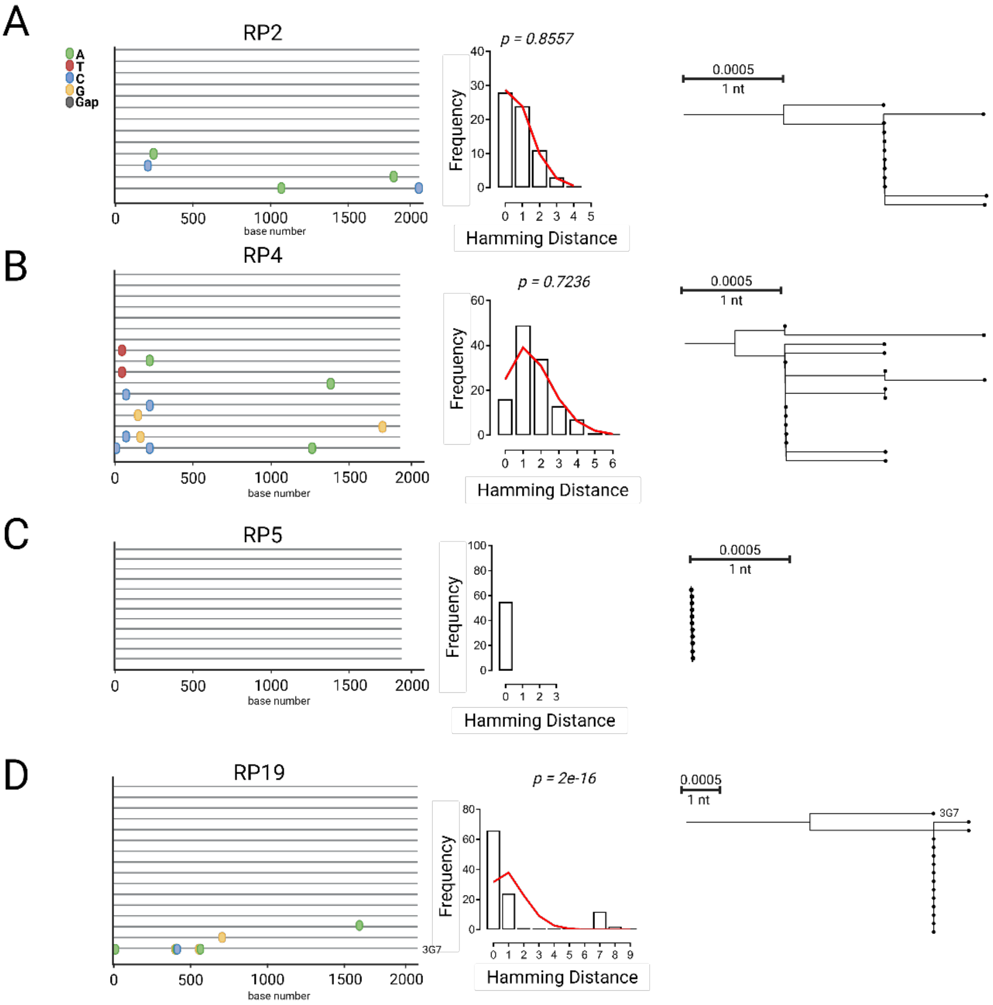

Fig. S5. Diversity of plasma *env* populations in group R5$_{\text{I-III}}$ (In addition to **Fig. 1**). Diversity of *env* sequences in plasma samples RP2, RP4, RP5, and RP19 are illustrated by *Highlighter* plots (left), HD frequency distributions (middle) and midpoint-rooted phylogenetic trees (right), as further detailed in the legend of **Fig. 1**. The data for RP2, RP4, RP5 (which contains only one haplotype), including HD frequencies that follow a Poisson distribution ($p > 0.05$), are consistent with an infection initiated by a single variant. In RP19, haplotype 3G7 contributed to the HD frequency distribution not obeying the Poisson distribution (see text for details).

Fig. S6

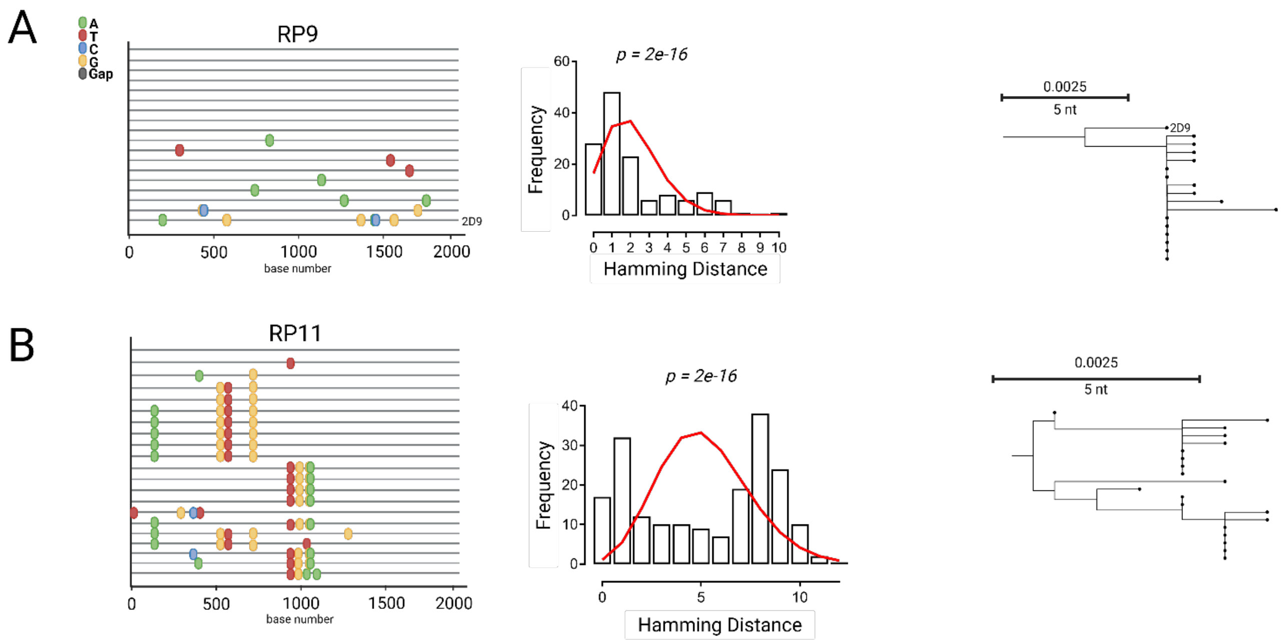

Fig. S6. Diversity of plasma *env* populations in group R5$_{IV-V}$ (In addition to **Fig. 2**). Diversity of *envs* in plasma samples RP9 and RP11 was analyzed as in Fig. 1 and Fig. S5. In RP9, one haplotype (2D9) contributed to deviation from the Poisson distribution of mutations. In RP11, as in RP6, the increased diversity in *envs* is uncertain as phylogenetic trees did not allow us to determine whether it reflected the onset of selective pressure or the transmission of multiple variants (see text for details).

<u>Fig. S7</u>

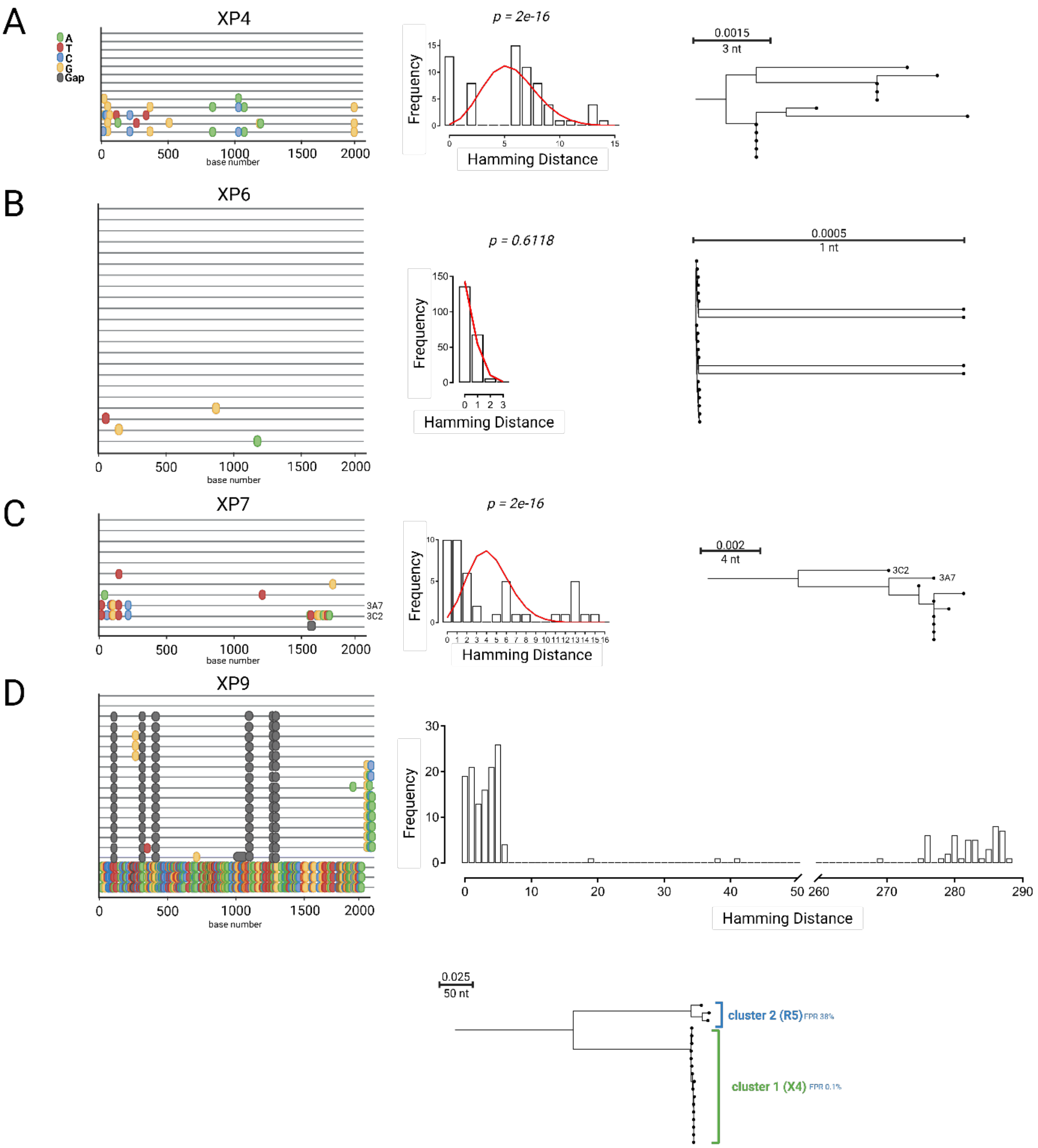

Fig. S7. Diversity of plasma *env* populations in group XP (In addition **to Fig. 3).** Diversity of *envs* in plasma samples XP4 **(A)**, XP6 **(B)**, XP7 **(C)**, and XP9 **(D)** was analyzed as in Figs. 1, 2, 3 and Figs. S5 and S6. XP4, like RP6 and RP11, showed increased *env* diversity, which may have resulted from immune pressure or the transmission of multiple variants. Data for XP6 are consistent with transmission of a single variant. XP7 contained two haplotypes that were enriched in mutations (3C2 and 3A7) but were clearly derived from other sequences in the sample, suggesting that XP7 also originated from transmission of a single sequence. In XP9, phylogenetic analysis indicated the presence of two distinct *env* clusters corresponding to R5 (cluster 2) and X4 (cluster 1) *envs*.

Fig. S8

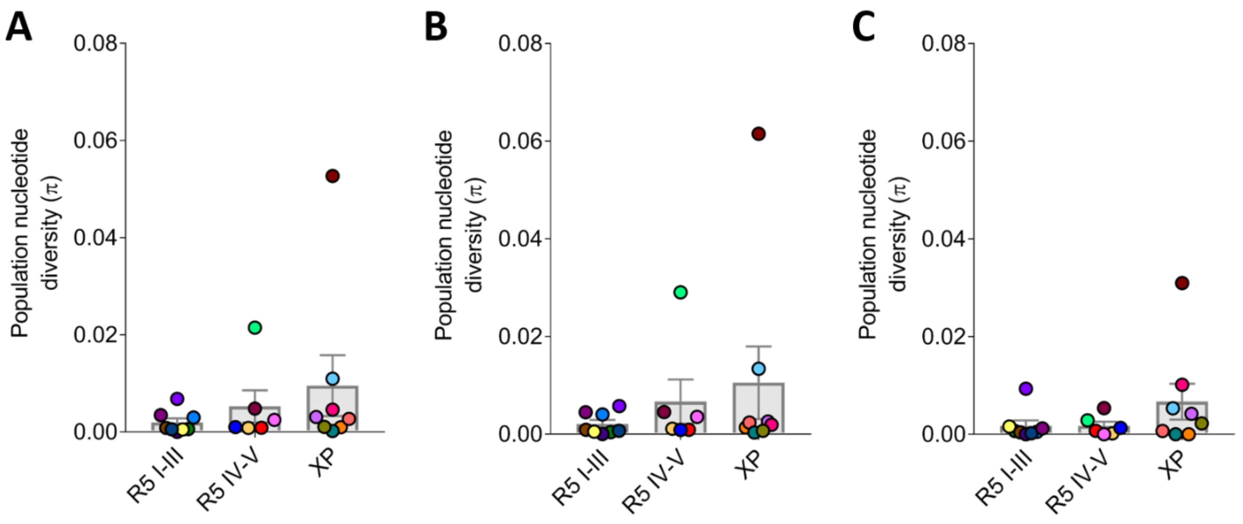

Fig. S8. Nucleotide diversity π_e of plasma *env* populations in groups R5$_{I\text{-}III}$, R5$_{IV\text{-}V}$ and XP. Values were calculated considering full length *envs* (A), gp120 (B) or gp41 (C) and are color coded as described in Fig. S4C. Means ± SEM are shown.

Fig. S9

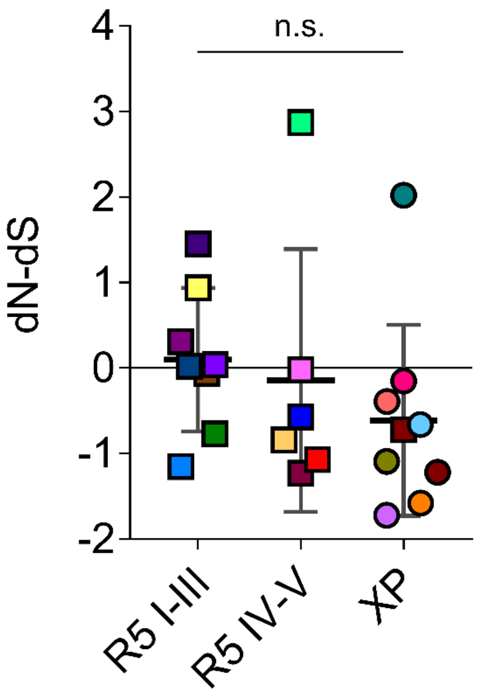

Fig. S9. Relative content of non-synonymous (dN) and synonymous (dS) mutations in plasma env sequences of groups R5$_{I-III}$, R5$_{IV-V}$ and XP. Differences between dN and dS in full length *envs* were calculated using MEGA11 with the Nei-Gojobori method and variance of the difference was computed using the bootstrap method (1000 replicates). Results represent means ± SD. Statistics: one-Way ANOVA followed by a Tukey's test.

Fig. S10

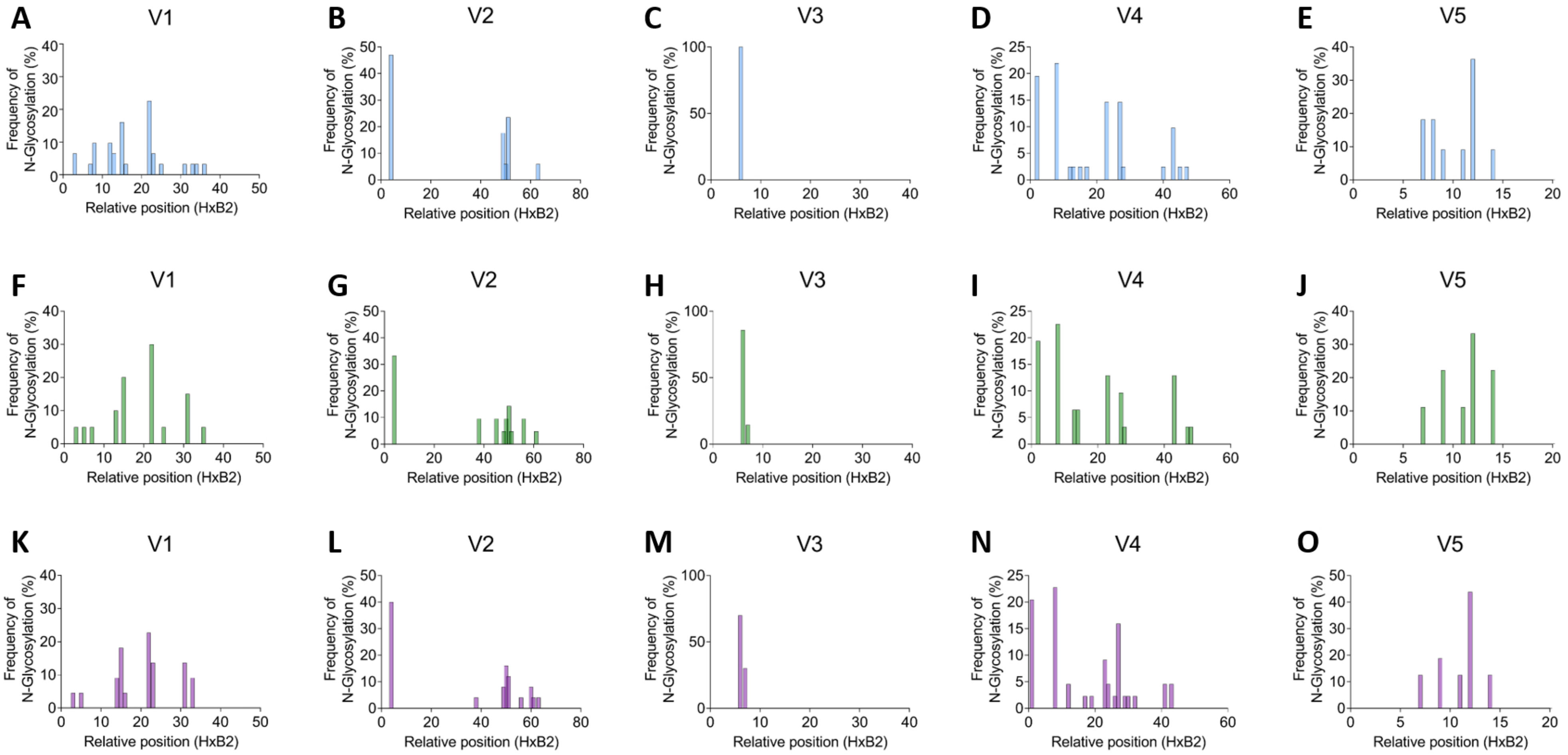

Fig. S10. Position frequency distribution of PNGS in gp120's variable loops. Consensus sequences of gp120 variable loops from groups $R5_{I\text{-}III}$ **(A-E)**, $R5_{IV\text{-}V}$ **(F-J)** and XP **(K-O)** were aligned with the HxB2_K03455 sequence and analyzed with the N-GlycoSite tool (www.hiv.lanl.gov) to determine the position of PNGS (N-X-S/T sequon). The number of PNGS at each amino acid position were then expressed in frequency (%) relative to the total number of sequences.
